# Cigarette Smoke Induces Canonical Stress Granule Formation in Human Bronchial Epithelial Cells in Reactive Oxygen Species- and PERK-Dependent Manners

**DOI:** 10.3390/biom16040615

**Published:** 2026-04-21

**Authors:** Mousumi Bhowmik, Chenkun Zheng, Bisrat Bekele, Jessica Failler, Carlie Klatt, Souren Farimani, Bryant Jones, Chung-Chun Tyan, Asmahan Abu-Arish

**Affiliations:** 1 Department of Anatomy, Physiology and Pharmacology, University of Saskatchewan, Saskatoon, SK S7N 5E5, Canada; ukc521@mail.usask.ca (M.B.); yiq173@mail.usask.ca (C.Z.); mzl610@mail.usask.ca (B.B.); jdp618@mail.usask.ca (J.F.); 2 Regina General Hospital, Regina Campus, University of Saskatchewan, Regina, SK S4P 0W5, Canada; cak278@mail.usask.ca; 3 Department of Neurology and Neurosurgery, Montreal Neurological Institute, McGill University, Montreal, QC H3A 2B4, Canada; souren.vahdatfarimani@mail.mcgill.ca; 4 School of Rehabilitation Science, University of Saskatchewan, Saskatoon, SK S7N 2Z4, Canada; bwj823@mail.usask.ca; 5 Division of Respirology, Critical Care and Sleep Medicine, University of Saskatchewan, Saskatoon, SK S7N 0W8, Canada

**Keywords:** cigarette smoke extract, human bronchial epithelial cells, reactive oxygen species, integrated stress response, eIF2α, PERK, stress granules, G3BP1, ISRIB, quantitative immunofluorescence imaging

## Abstract

Cigarette smoke (CS) is the primary risk factor for the development of chronic obstructive pulmonary disease (COPD). Investigating the impact of CS on human airway epithelium is important for understanding COPD development and combating its effects. While some studies show that long exposure to CS activates inflammasome formation in airway epithelium, leading to cytokines’ maturation and release, its acute effect on inflammation regulation requires further elucidation. Due to the importance of acute cellular responses in modulating cell survival and controlling inflammatory outcomes, we examined the effect of acute cigarette smoke extract exposure on human bronchial epithelial cells. Due to the high reactive oxygen species content in CS, we hypothesize that acute CS exposure activates the integrated stress response (ISR) pathway leading to stress granules (SG) formation to facilitate oxidative stress resolution and promote cell survival. Immunostaining, fluorescence confocal imaging, quantitative analyses, and immunoblotting were performed to test our hypothesis. We report here that acute exposure to CS extract triggers canonical SG formation by activating the ISR pathway via the PERK/eIF2α arm in a reactive oxygen species-dependent manner. SG formation is abolished upon inhibiting PERK or eIF2α function, or by scavenging oxidants prior to smoke exposure. Characterizing SG formation in terms of measuring SG size and abundance and the sequestration of the SG marker G3BP1 reveals that SG formation is maximal at 15% CS extract exposure for 2 h and undergoes gradual disassembly at longer exposure times. This is closely dependent on cytoplasmic p-eIF2α levels. These results demonstrate that acute exposure to CS activates the protective ISR pathway to potentially reduce the detrimental effects of CS and promote stress resolution and cell survival.

## 1. Introduction

Chronic obstructive pulmonary disease (COPD) is a leading cause of hospitalization and death in Canada [1,2] and cigarette smoke is the primary risk factor in its development and is responsible for most cases [3,4]. Yet the molecular mechanisms responsible for COPD pathogenesis resulting from cigarette smoke exposure remain unknown. Smoke induces functional downregulation of the cystic fibrosis transmembrane conductance regulator (CFTR) ion channel [5,6,7,8,9,10,11,12,13] which in turn dysregulates the homeostatic inflammatory responses of the airway, mimicking cystic fibrosis (CF) [14].

Prolonged exposure to cigarette smoke (24 h) has been reported to induce NLRP3 or AIM2 inflammasomes activation in macrophages and human bronchial epithelial cells [15,16,17,18,19]. Inflammasomes are cytoplasmic complexes composed of various proteins that, when assembled, become active and induce the maturation and secretion of pro-inflammatory cytokines such as IL-1β [20,21]. Secreted cytokines then amplify the response of the immune system by enhancing the infiltration of immune cells and the release of additional cytokines. Yet, the acute response of human airway epithelium to smoke exposure is not well characterized.

Cigarette smoke contains a high concentration of reactive oxygen species (ROS) [8,22]. ROS consume oxygen, oxidize proteins, lipids, and DNA, and deplete the natural antioxidant defenses in the lung, thereby increasing oxidative stress [23,24]. To protect against oxidative stress, cells activate mechanisms such as the integrated stress response (ISR) [25]. This mechanism is triggered by activating the phosphorylation of one of four mammalian kinases in response to various stressors [26]; namely protein kinase R (PKR), PKR-like endoplasmic reticulum kinase (PERK), general control nonderepressible 2 (GCN2) and heme-regulated inhibitor (HRI) [27,28,29]. Oxidative stress due to the enrichment of ROS in smoke [8,22,24,30] may result in the phosphorylation of PKR and GCN2 [25,26,27,28,29,31,32,33]. When activated, these kinases in turn phosphorylate the eukaryotic initiation factor 2α (eIF2α) at Ser51, which initiates the formation of stress granules (SGs) to halt general protein translation by sequestering untranslated mRNAs [26]. Early ISR (1–3 h) enables cells to effectively cope with diverse stressful conditions by suppressing general protein synthesis while upregulating ATF4 protein expression to induce the generation of antioxidants [26]. During late ISR (~6 h), phosphatases such as protein phosphate type 1 (PP1) and its regulatory subunits GADD34 and CReP are upregulated [26,34], leading to eIF2α dephosphorylation to reinstate protein synthesis and cell homeostasis [35]. It is not known whether acute cigarette smoke exposure induces SG formation to promote cell survival.

Due to the importance of acute cellular responses in modulating cell survival and controlling inflammatory outcomes, we examined the effect of short-term cigarette smoke exposure (1–6 h) on human bronchial epithelial cells. Intriguingly, our results show that the ISR pathway is indeed activated in response to acute cigarette smoke extract exposure by phosphorylating the PERK/eIF2α axis leading to the formation of numerous SGs. The ability of the ROS scavenger N-acetylcysteine (NAC) to block SG formation and halt the increase in both p-eIF2α and p-PERK abundance following smoke exposure further supports an essential role for ROS in this response. Our results support a role for cigarette smoke-associated ROS in acutely stimulating the ISR pathway to induce cell survival by inducing SG formation.

## 2. Materials and Methods

CFBE Cell culture: The human cystic fibrosis bronchial epithelial (CFBE) cell line expressing WT-CFTR [6,36,37] was a kind gift from Dr. Lianwu Fu at the University of Alabama at Birmingham. Cells were maintained at 37 °C, 5% CO_2_, and 95% humidity in eagle’s minimum essential medium (EMEM) supplemented with 10% Fetal bovine serum (FBS), 1% L-glutamine (2 mM), and 1% penicillin–streptomycin. Cells were cultured every 5 days at 90–100% confluency. Cells were mostly passaged up to 15 times.

For immunofluorescence (IF) imaging experiments, 50,000 cells were seeded on collagen coated glass-bottom MatTek dishes (no. 1.5) for 48 h before treatment. For immunoblotting experiments, 120,000 cells were seeded in 6-well culture plates for 72 h before treatment. Media was refreshed 18 h after cell seeding. Cells were gently starved in OptiMEM for 18 h prior to treatment and/or smoke exposure to induce a state of dormancy.

Primary human bronchial epithelial cells and tissue: The primary human bronchial epithelial tissue was procured from the bronchi of donors who underwent bronchoscopy for diagnostic purposes other than this study. Informed consent for participation was obtained from all donors. This procedure is routinely performed by Dr. Chung-Chun Tyan at the Royal University Hospital in Saskatoon, SK, Canada. The study was conducted in accordance with the Tri-Council Policy Statement: Ethical Conduct for Research Involving Humans—TCPS 2 (2022), and the protocol (Certificate ID: Bio 2943) was approved by the University of Saskatchewan Biomedical Research Ethics Board on 16 January 2025.

Some of the sourced tissue was immediately divided into two tubes and exposed to smoke extract or HBSS, then cyto-spun on coated cytology slides (Epredia, Kalamazoo, MI, USA), fixed and immunoassayed as detailed under the immunofluorescence section. Tissue samples were mounted in ProLong^TM^ Diamond Antifade Mountant (Thermofisher Scientific, Waltham, MA, USA). Other tissue was immediately seeded on collagen-coated glass-bottom MatTek dishes for 8–12 days to achieve cell de-differentiation so cells resemble basal CFBE cells. The culture was maintained in bronchial epithelial growth media (BEGM) purchased from the CFTRc at McGill University. Upon achieving 80% confluency, the cell culture was exposed to smoke extract or HBSS and processed as detailed under the immunofluorescence section. Tissue samples procured from three bronchoscopy donors were used for each type of experiment. Tissue procurement and processing are reviewed and approved by the Human Ethics Review Board at the University of Saskatchewan and are in line with all guidelines and regulations at the University of Saskatchewan.

Reagents: Fetal bovine serum (FBS), EMEM, L-glutamine, penicillin–streptomycin, Hank’s balanced salt solution (HBSS), and phosphate-buffered saline (PBS) were purchased from Wisent Inc (Saint-Jean-Baptiste, QC, Canada). ISRIB, GSK2606414, C16 and NAC were purchased from Sigma-Aldrich (St. Louis, MO, USA). OptiMEM, DAPI and DMSO were purchased from ThermoFisher Scientific.

Cigarette smoke extract preparation: The standardized research-grade 1R6F cigarettes were purchased from the Center for Tobacco Reference Products at the University of Kentucky (Lexington, KY, USA). Cigarettes were routinely stored at 4 °C for ultimate freshness. Before extract preparation, cigarettes and saline solution (HBSS) were conditioned at room temperature for 30 min. The extract was prepared immediately before cell exposure using the method in Carp et al. [38]. Briefly, one cigarette was gently puffed for 2 min in room temperature HBSS (10 mL) using a vacuum system. The extract was not filtered to maintain potency and ensure the coexistence of the gas and tar phases of cigarette smoke in the prepared extract to replicate smoking more closely. The extract concentration was immediately measured (within 5–10 min) using a spectrophotometer (Bio-Rad, Hercules, CA, USA) with an optical density of 0.65 at 320 nm absorption wavelength corresponding to 100%. An optical density of 0.8–1.2 was consistently achieved. Cells were exposed to the extract within 20 min after preparation.

Treatments: At 60–70% confluence, cells were exposed to different cigarette smoke extract concentrations (7.5, 15, 30 and 50%) for a set duration (1, 2, 4 and 6 h). In some experiments, cells were pretreated with ISRIB (200 nM, 1 h), GSK2606414 (2 µM, 1 h), C16 (1 µM, 1 h) or N-acetylcysteine (NAC, 5 mM, 15 min) prior to smoke extract exposure, and these treatments were replenished upon extract exposure.

Antibodies: Rabbit polyclonal phospho-PERK antibody (ThermoFisher Scientific), rabbit monoclonal phospho-eIF2α antibody (Cell Signaling Technology, Boston, MA, USA) and mouse monoclonal G3BP1 antibody (Santa Cruz Biotechnology, Inc., Dallas, TX, USA) were used for immunofluorescence and immunoblotting experiments. The hFAB™ Rhodamine β-Actin and GAPDH primary antibodies were used for immunoblotting experiments (Bio-Rad).

Immunofluorescence (IF): The IF protocol was adapted from Abu-Arish et al. [39]. Briefly, cells were quickly washed twice in 37 °C PBS before fixation in 10% buffered formalin for 15 min. Cells were then permeabilized using 0.5% Triton X-100/PBS for 15 min, blocked with 2% BSA/PBS for 1 h and incubated overnight at 4 °C with primary antibodies. They were then exposed to Alexa Fluor 488 goat anti-mouse and Alexa Fluor 633 goat anti-rabbit secondary antibodies (1:1000 dilution; Invitrogen, Carlsbad, CA, USA) for 1 h at room temperature, exposed to DAPI for 3 min to label cell nuclei, washed, and mounted in PBS for imaging. DAPI fluorescence signal was used to mark cell nuclei to segregate nuclear and cytoplasmic fluorescence signals in a region of interest (ROI) during quantitative nucleus-based image analysis.

Immunoblotting: Immunoblotting protocol was adapted from Abu-Arish et al. [39]. Briefly, at ~85% confluence, cells were serum starved in OptiMEM medium (5% CO_2_/95% air, 37 °C) for 18 h. Cells were then treated with ISRIB, GSK, NAC, C16 or vehicle control before exposure to HBSS or smoke extract for the intended duration. Cultures were rinsed twice with ice-cold PBS. Scraped cells were centrifuged at 4 °C and cell pellets were solubilized in ice-cold lysis buffer (150 mM NaCl, 20 mM Tris, 0.08% *w*/*v* deoxycholic acid, 1% *w*/*v* Triton X-100, 0.1% SDS, and protease/phosphatase inhibitors (ThermoFisher Scientific), pH-8) for 30 min at 4 °C. Total protein content was measured using a Pierce^TM^ BCA Protein Assay Kit (ThermoFisher Scientific). Next, 2× Laemmli sample buffer (Bio-Rad) with 355 mM 2-mercaptoethanol was added at a 1:1 ratio and samples were heat-shocked at 95 °C for 5 min. Cells and lysates were kept on ice and solutions were prechilled to 4 °C. SDS-PAGE was performed using 4–20% Mini-PROTEAN^®^ TGX™ Precast Protein Gels (Bio-Rad). Proteins were transferred to supported nitrocellulose membranes (0.22 pore size; Bio-Rad) for immunoblotting. Membranes were blocked with 5% BSA TTBS for 1 h and incubated overnight at 4 °C with primary antibodies. Exposure to secondary antibodies, goat anti-rabbit horseradish peroxidase (HRP) or goat anti-mouse HRP (1:10,000 dilution; Invitrogen), was carried out for 2 h at room temperature. Protein signals were acquired using the Bio-Rad Chemidoc imaging system and Image Lab^TM^ (Bio-Rad) was used for analysis. Measurements were normalized to the β-Actin or GAPDH protein signals.

**Figure 1:**
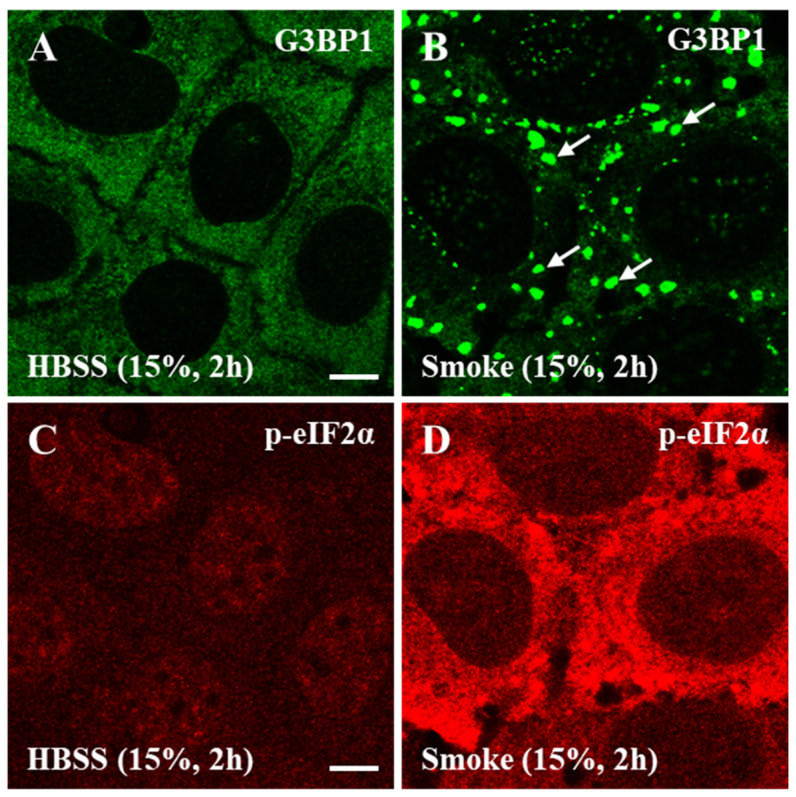
Human bronchial epithelial cells respond to acute cigarette smoke extract exposure by forming canonical stress granules. CFBE cells were exposed to 15% HBSS (vehicle control) or 15% smoke extract for 2 h. Immunofluorescence (IF) and fluorescence confocal imaging (hereafter referred to as IF imaging) of endogenous G3BP1 and p-eIF2α distributions and levels were performed to visualize SG formation under smoke exposure condition in relation to eIF2α phosphorylation. (**A**) IF imaging of a region of interest (ROI) encompassing several CFBE cells focused at cells mid-section showed that G3BP1 (green) is distributed homogenously in the cell cytoplasm under HBSS conditions and (**B**) that exposure to smoke extract stimulated G3BP1 redistribution into stress granules (SGs, white arrows). To establish the canonicality of SG formation under smoke exposure conditions in the same cells as in (**A**,**B**), IF imaging visualized the endogenous distribution and abundance of phosphorylated eIF2α (p-eIF2α, red). (**C**) Cytoplasmic p-eIF2α abundance was low under HBSS condition and (**D**) markedly increased in the cell cytoplasm following smoke exposure. IF imaging demonstrated that SG formation under smoke exposure condition was associated with a significant increase in cytoplasmic p-eIF2α abundance, suggesting canonicality. Confocal image scale bar = 7.7 µm. Abbreviations: SG = stress granule, ROI = region of interest, and IF = immunofluorescence.

We have discovered a relationship between the number of cells seeded per MatTek dish, the volume of cell media used, and amount of smoke extract added. For example, upon doubling the number of cells without doubling the media, exposure to 15% smoke extract for 2 h did not induce strong SG formation. Instead, doubling the smoke extract (30%) while keeping the cell media constant resulted in the usual SG formation seen in Figure 1B. Alternatively, keeping the smoke extract volume constant (15%) while doubling the cell media volume also resulted in SG formation like that in Figure 1B. We therefore concluded that the amount of smoke/cell must remain constant to achieve similar SG formation. We applied this concept in immunoblotting experiments where we compensated for the increase in the number of cells by proportionally increasing the cell media volume and the used smoke extract volume. So, to achieve the same effect as in immunofluorescence imaging experiments where we expose cells to 15% smoke extract for 2 h, immunoblotting experiments dictated the use of 30% smoke extract for 2 h and doubling the volume of cell media.

Confocal fluorescence imaging: Fluorescence confocal images were collected using the LSM 700 (Zeiss, Oberkochen, Germany) and the Stellaris 8 (Leica, Wetzlar, Germany) confocal microscopes; a 63× (NA = 1.4) oil immersion objective was used. Images were collected at the following conditions: image size of 1024 × 1024 pixel, pixel size of 0.1 µm (LSM 700) and 0.06 µm (Leica) and pixel dwell time of 12 µs (LSM 700) and 4 µs (Leica). Six (LSM 700) or ten (Leica) ROIs were collected per experiment. Laser powers were optimized to eliminate fluorescence saturation and minimize fluorescence photobleaching while maximizing fluorescence signal. A protein of interest was imaged under similar imaging conditions under different treatment conditions.

Quantitative image analysis: The mean and cumulative fluorescence intensity in a fluorescence confocal image were measured using two analyses: cluster and nucleus-based image analyses. Both analyses are fluorescence intensity-threshold-based, semi-automated and coded using the widely used ImageJ software (1.54p).

The nucleus-based image analysis takes advantage of DAPI fluorescence signal to accurately define and mask all nuclei in a confocal image (ROI), which facilitates the segregation of the fluorescence intensity of the nuclear and cytoplasmic compartments in an ROI. This is accomplished by subtracting the integrated (cumulative) fluorescence intensity of the combined nuclear contributions (from several nuclei) from the total cumulative fluorescence intensity of an ROI. The mean cytoplasmic fluorescence intensity of a protein of interest is then calculated by dividing the cumulative cytoplasmic fluorescence intensity by the cytoplasmic area (in µm^2^). For accuracy, the analysis dictates that every ROI is fully occupied by cells to reduce noise and errors when calculating the cytoplasmic fluorescence contribution. This analysis is used in this study to calculate the mean cytoplasmic p-eIF2α and cumulative cytoplasmic G3BP1 abundance under various treatment conditions.

The cluster analysis takes advantage of the strong G3BP1 fluorescence signal under SG formation condition to define and mask SGs in a similar fashion as the nucleus-based image analysis. A watershed mask is used to separate adjacent SGs for more accurate measurement of SG numbers. The analysis counts the number of SGs, measures their individual size in µm^2^ and measures the integrated (cumulative) or mean fluorescence intensity of a protein of interest encompassed in each cluster. All the above functions are provided by the ImageJ software.

Statistical tests: GraphPad Prism 10 was used to perform all statistical analyses and data visualization. No pairing or matching were assumed. If distribution normality or lognormality was not achieved, nonparametric statistical tests were performed as detailed in the figure legends. The nonparametric Kruskal–Wallis test was performed for most analyses and the recommended Dunn’s test was used to correct for multiple comparisons. Outliers were identified using the ROUT method with Q = 1%. All *p* values are also reported in figure legends.

## 3. Results

### 3.1. Human Bronchial Epithelial Cells and Tissue Respond to Acute Cigarette Smoke Extract Exposure by Forming Stress Granules

We first hypothesized that acute cigarette smoke (henceforth referred to as smoke for simplicity) exposure activates the ISR pathway and the formation of SGs. To examine this, human bronchial epithelial cells, namely the CFBE cell line expressing wild type CFTR, were exposed to 15% HBSS (vehicle control) or smoke extract for 2 h. The localization and distribution of endogenous Ras GTPase-activating protein-binding protein 1 (G3BP1) protein, an essential SG marker, was then visualized using immunofluorescence (IF) staining and fluorescence confocal imaging (henceforth referred to as IF imaging). IF imaging showed that G3BP1 is homogenously distributed in cell cytoplasm under HBSS conditions (Figure 1A) and aggregated into irregularly shaped clusters following acute smoke exposure (Figure 1B, white arrows). These cytoplasmic clusters resembled SGs formed in response to sodium arsenite treatment in other cell types [40]. IF imaging evidently demonstrated SG formation under acute smoke exposure conditions, partially validating our hypothesis. To the best of our knowledge, this is the first report of SG formation in human bronchial epithelial cells in response to smoke exposure or other stressors.

**Figure 2:**
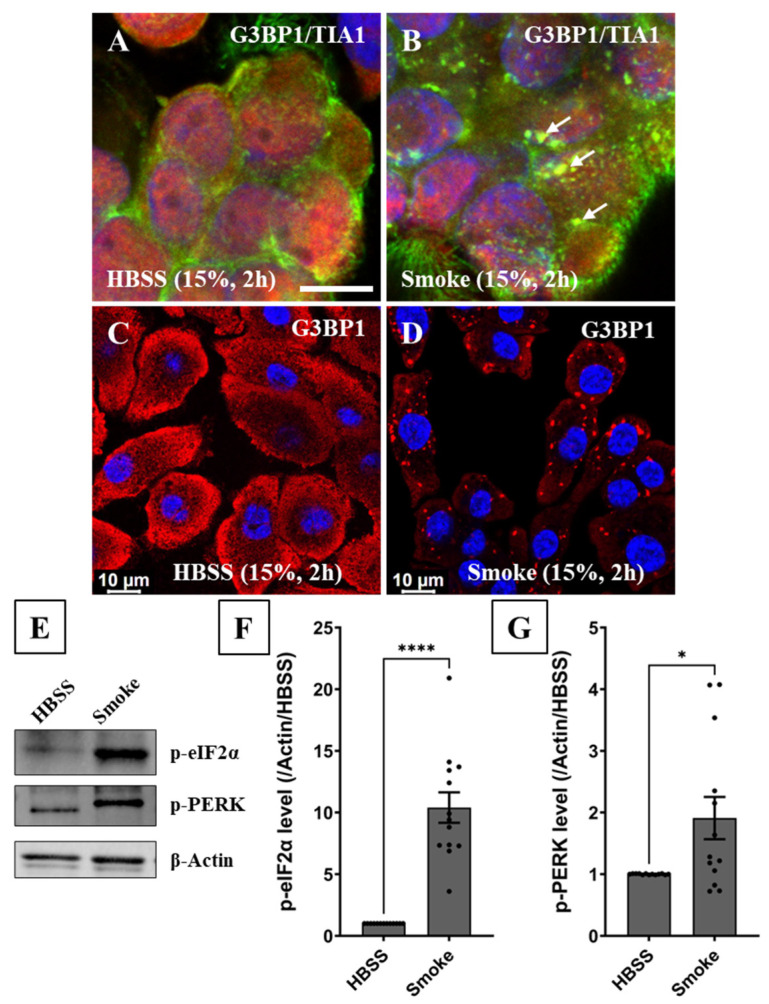
Primary human bronchial epithelial cells and tissue respond to acute cigarette smoke extract exposure by forming canonical stress granules like CFBE cells. Primary human bronchial epithelial cells and tissue isolated from the bronchi of consented donors via bronchoscopy were exposed to 15% HBSS (vehicle control) or 15% smoke extract for 2 h. IF imaging of endogenous G3BP1 distribution and immunoblotting of p-eIF2α and p-PERK abundance were performed to demonstrate canonical SG formation under smoke exposure conditions. (**A**) IF imaging of an ROI encompassing procured and immunoassayed human bronchial epithelial tissue showed that SG markers G3BP1 (green) and TIA1 (red) were distributed homogenously in the cell cytoplasm under HBSS conditions and (**B**) that exposure to smoke extract stimulated their aggregation and colocalization into SGs (white arrows). (**A**,**B**) Confocal image scale bar = 7.5 µm. (**C**,**D**) SG formation in response to smoke extract exposure was also visualized in the cytoplasm of basal (de-differentiated) primary human bronchial epithelial cells as marked by G3BP1 aggregation (red). The immunofluorescence imaging experiments were reproduced in human bronchial epithelial cells isolated from 4 bronchoscopy donors. Confocal image scale bar = 10 µm. (**E**–**G**) To establish canonicality of SG formation in primary human bronchial epithelial cells isolated from bronchoscopy donors, p-eIF2α levels were measured using immunoblotting under HBSS and smoke exposure conditions. (**E**) Smoke exposure markedly increased p-eIF2α and p-PERK levels, assuring us that SG formation is canonical in a PERK phosphorylation-dependent manner (see Figure S2). (**F**) Immunoblotting analysis measured a 10-fold increase in p-eIF2α levels in response to smoke exposure and (**G**) a 2-fold increase in p-PERK levels. Immunoblotting was performed using human bronchial epithelial cells isolated from 6 bronchoscopy donors with two technical replicates per donor. Welch’s test was performed with *: *p* = 0.02 and ****: *p* < 0.0001. Abbreviations: SG = stress granule, ROI = region of interest, and IF = immunofluorescence. Western blot original images can be found in Supplementary Materials.

To ensure the translatability and validity of our findings in CFBE cells, primary human bronchial epithelial tissue freshly procured from donors’ bronchi (via bronchoscopy by bronchoscopist Dr. Tyan) was exposed to 15% smoke extract or vehicle control for 2 h, cyto-spun on coated slides and immunoassayed (Figure 2A,B). The sourced tissue was also cultured on collagen-coated, glass-bottom dishes for 8–12 days to promote de-differentiation to resemble basal cells and then exposed to 15% smoke extract for 2 h (Figure 2C,D). Cells maintained under both conditions robustly formed SGs in response to smoke exposure, validating our findings in CFBE cells and assuring us that smoke-induced SG formation is a conserved phenomenon in both primary human bronchial epithelial cells and tissue.

### 3.2. Characterizing Stress Granule Formation in Response to Increasing Smoke Concentrations and Exposure Times

**Figure 3:**
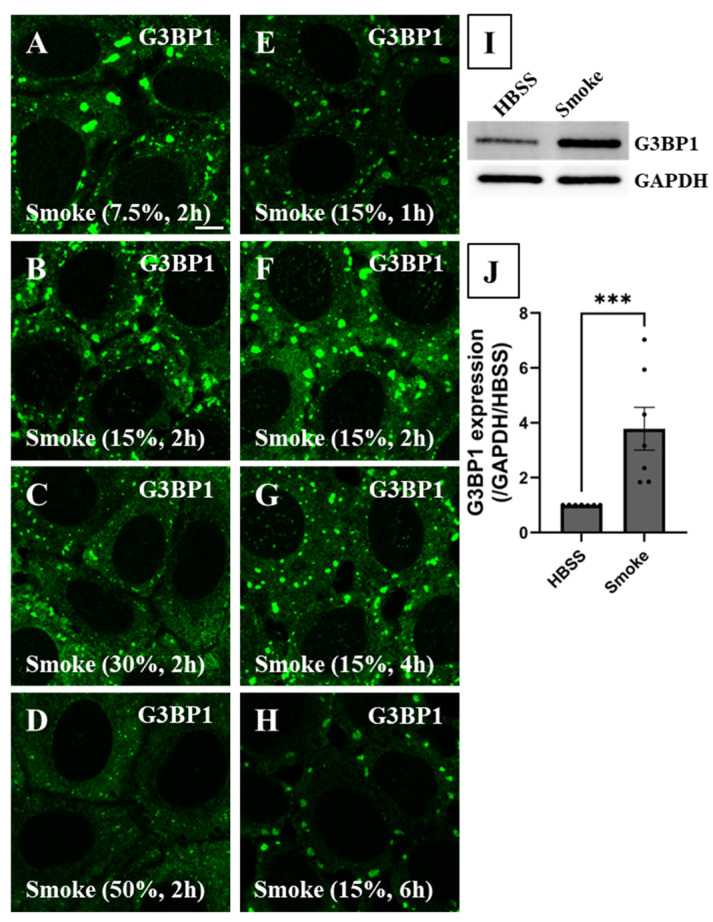
SG formation visualization in response to different smoke extract concentrations and exposure times. CFBE cells were exposed to a wide range of smoke extract concentrations for different exposure times to characterize the optimal SG formation conditions. IF imaging was used to visualize SG formation as marked by G3BP1 aggregation (green) under various smoke exposure conditions while cellular G3BP1 abundance was measured using immunoblotting analysis. (**A**–**D**) Cells were exposed to increasing smoke extract concentrations (7.5, 15, 30, 50%) for 2 h and SG formation was assessed by visualizing endogenous G3BP1 distribution using IF imaging. IF imaging showed that SG formation took place under 7.5% smoke extract concentration. The largest SGs formed under 15% smoke extract condition and significantly smaller SGs were observed under 30 and 50% smoke extract concentration conditions. (**E**–**H**) SG formation was then assessed under 15% smoke extract concentration at various exposure times (1, 2, 4, 6 h). SG formation peaked at 1 to 2 h and decreased at 4 and 6 h post-exposure, suggesting disassembly. Each ROI is an independent biological sample. (**A**–**H**) Confocal image scale bar = 7.7 µm. (**I**,**J**) Immunoblotting demonstrated a significant increase in G3BP1 abundance following exposure to smoke extract (N = 7). See also Figure S3. Th nonparametric Kolmogorov–Smirnov test was performed with ***: *p* = 0.0006. Abbreviations: SG = stress granule, IF = immunofluorescence, ROI = region of interest, and N = total number of independent experiments. Western blot original images can be found in Supplementary Materials.

To determine the optimal SG maturation conditions, we then characterized SG formation in response to a range of smoke extract concentrations (7.5–50%) and exposure times (1–6 h) in CFBE cells using a quantitative cluster-based image analysis. The cluster analysis counts G3BP1 clusters (#SGs) per region of interest (ROI, such as the confocal image in Figure 3A) and measures SG size in µm^2^. The analysis also measures the mean G3BP1 fluorescence intensity per SG averaged over all SGs in an ROI, the cumulative (integrated) G3BP1 fluorescence intensity per SG averaged over all SGs in an ROI, and the cumulative SG G3BP1 fluorescence in an ROI by summing all cumulative G3BP1 fluorescence intensities in all SGs in an ROI. The latest measurement represents the total sequestered G3BP1 in all SGs.

**Figure 4:**
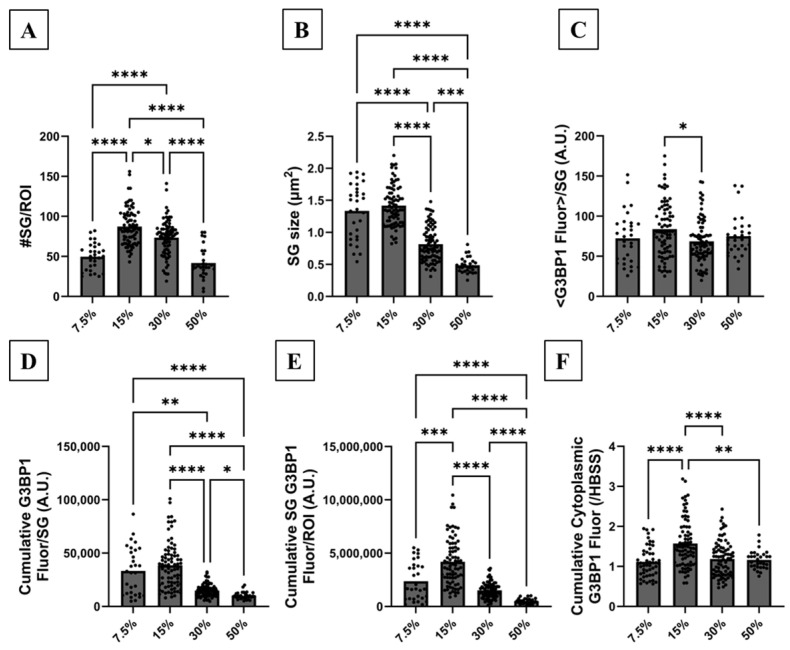
SG formation quantification in response to different smoke extract concentrations. CFBE cells were exposed to various smoke extract concentrations (7.5, 15, 30, 50%) for 2 h. IF imaging and quantitative cluster analysis were performed to measure the total number of SGs per ROI, SG size in µm^2^, mean G3BP1 fluorescence per SG, cumulative G3BP1 fluorescence per SG and total cumulative G3BP1 fluorescence per SG for all SGs in an ROI. SGs did not form under 0% smoke extract exposure and thus no cluster analysis was performed. Exposure to 7.5% smoke extract resulted in SG formation in 3 out of 5 experiments. Exposure to 15% smoke extract concentration resulted in the formation of the largest and most abundant SGs. (**A**) Cluster analysis showed that exposure to 15% smoke extract for 2 h resulted in maximal SG numbers per ROI, which gradually and significantly decreased under 30 and 50% smoke extract exposure (#SG_7.5%_ = 50 ± 3, #SG_15%_ = 87 ± 3, #SG_30%_ = 73 ± 2, #SG_50%_ = 42 ± 4; N_0%_ = 8, n_0%_ = 80; N_7.5%_ = 3, n_7.5%_ = 30; N_15%_ = 8, n_15%_ = 80; N_30%_ = 8, n_30%_ = 80; N_50%_ = 3, n_50%_ = 29 (1 outlier was identified and excluded)). (**B**) Cluster analysis also demonstrated that exposure to 15% smoke extract for 2 h resulted in the formation of the largest and most mature SGs with average size = 1.42 µm^2^. SG size significantly decreased under 30 and 50% smoke extract exposure (Size_7.5%_ = 1.33 ± 0.08, Size_15%_ = 1.42 ± 0.04, Size_30%_ = 0.82 ± 0.03, Size_50%_ = 0.49 ± 0.02 µm^2^; N_0%_ = 8, n_0%_ = 80; N_7.5%_ = 3, n_7.5%_ = 30; N_15%_ = 8, n_15%_ = 80; N_30%_ = 8, n_30%_ = 80; N_50%_ = 3, n_50%_ = 30). (**C**) Mean G3BP1 fluorescence per SG was calculated and averaged over all SGs per ROI under the above conditions. The mean fluorescence level was not drastically different under all smoke extract concentrations with exposure to 15% smoke extract resulting in a higher mean fluorescence (N_7.5%_ = 3, n_7.5%_ = 30; N_15%_ = 8, n_15%_ = 80; N_30%_ = 8, n_30%_ = 80; N_50%_ = 3, n_50%_ = 30). (**D**) The cumulative G3BP1 fluorescence per SG was measured and averaged over all SGs in an ROI under the above conditions. This measurement encompasses the mean G3BP1 fluorescence per SG and SG size. The maximal cumulative fluorescence level per SG was achieved under 7.5 and 15% smoke extract exposure for 2 h and decreased gradually and significantly under 30 and 50% smoke exposure conditions (N_7.5%_ = 3, n_7.5%_ = 30; N_15%_ = 8, n_15%_ = 80; N_30%_ = 8, n_30%_ = 78 (2 outliers were identified and excluded); N_50%_ = 3, n_50%_ = 30). (**E**) The sum of cumulative G3BP1 fluorescence per SG in an ROI encompasses the mean G3BP1 fluorescence and SG size and abundance, which reflects the entire G3BP1 population sequestered into SGs per ROI. Exposure to 15% smoke extract resulted in maximal G3BP1 segregation into SGs which gradually and significantly decreased under 30 and 50% smoke extract concentrations suggesting immature formation (N_7.5%_ = 3, n_7.5%_ = 30; N_15%_ = 8, n_15%_ = 80; N_30%_ = 8, n_30%_ = 78 (2 outliers were identified and excluded); N_50%_ = 3, n_50%_ = 29 (1 outlier was identified and excluded)). (**F**) Using nucleus-based image analysis, cumulative cytoplasmic G3BP1 (including sequestered G3BP1 into SGs) was most abundant under 15% smoke extract exposure condition and decreased at higher smoke extract concentrations (N_7.5%_ = 5, n_7.5%_ = 50; N_15%_ = 8, n_15%_ = 80; N_30%_ = 8, n_30%_ = 80; N_50%_ = 3, n_50%_ = 30). The nonparametric Kruskal–Wallis test was used to calculate significance with *: *p* < 0.018, **: *p* < 0.0022, ***: *p* < 0.0006 and ****: *p* < 0.0001. All other comparisons are not significantly different. Each ROI is an independent biological sample. Data are presented as mean ± SEM. Abbreviations: SEM = standard error of mean, SG = stress granule, ROI = region of interest, N = total number of independent immunofluorescence experiments, n = total number of technical replicates (or analyzed ROIs) in all immunofluorescence experiments (n = N × number of technical replicates per experiment) and #SG for total number of SGs per ROI.

Cells were initially exposed to 7.5, 15, 30 and 50% smoke extract for 2 h and immunoassayed to characterize SG formation by targeting G3BP1 distribution as shown in Figure 3A–D. The choice of 2 h exposure time was based on preliminary experiments that are not discussed in this paper. When cells were exposed to 7.5% smoke extract, SGs formed in three out of five experiments. This suggests that this concentration borders SG formation and a slight change in smoke extract potency can tip the SG formation scale in one direction or another. Visual inspection and cluster analysis demonstrated that cells exposed to 15% smoke extract formed the most abundant and largest SGs (Figure 3B and Figure 4A). Cells responded to higher smoke concentrations (30 and 50%) by forming significantly fewer and smaller SGs (Figure 3C,D and Figure 4A,B), suggesting that excessive stress hinders SG formation. The molecular factor responsible for impairing SG formation at higher smoke concentrations is discussed in the next section. The mean G3BP1 fluorescence level per SG, on the other hand, remained mostly unchanged (Figure 4C), suggesting that it is not a determining variable in SG formation under these conditions. Cumulative G3BP1 fluorescence per SG is proportional to mean G3BP1 fluorescence per SG and SG size. Therefore, while the mean G3BP1 shows little variance, larger SGs result in higher cumulative fluorescence measurement. Exposure to 15% smoke extract resulted in maximal cumulative G3BP1 fluorescence per SG, which gradually and significantly decreased under 30 and 50% smoke extract exposure condition (Figure 4D). The total cumulative G3BP1 fluorescence for all SGs in an ROI is proportional to mean G3BP1 fluorescence and SG number and size. Cluster analysis showed that this parameter was greatest under 15% smoke exposure condition (Figure 4E) and significantly decreased under 30 and 50% smoke exposure conditions. Taken together, optimal SG formation and G3BP1 sequestration in human bronchial epithelial cells were achieved under 15% smoke extract exposure for 2 h.

**Figure 5:**
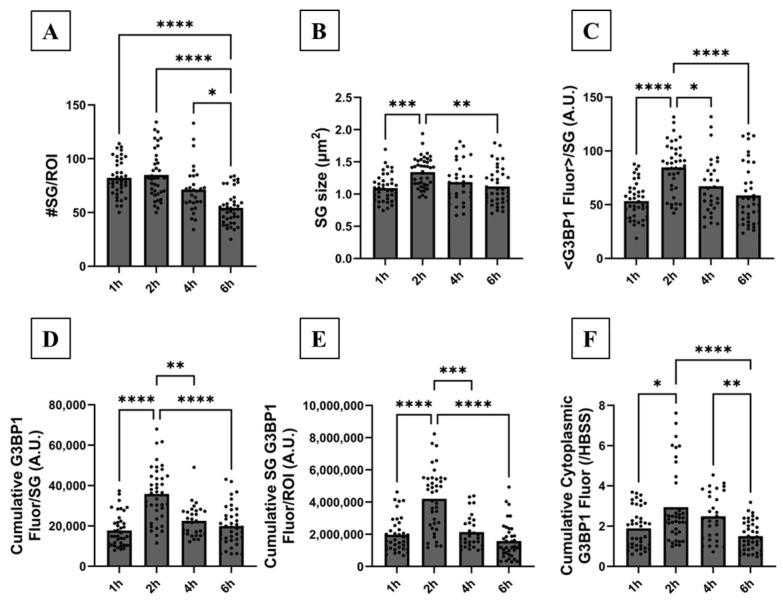
SG formation quantification in response to different smoke extract exposure times. CFBE cells were exposed to 15% smoke extract for various exposure times (1, 2, 4, 6 h). IF imaging and quantitative cluster analysis were performed to measure the total number of SGs per ROI, SG size in µm^2^, mean G3BP1 fluorescence per SG, cumulative G3BP1 fluorescence per SG and total cumulative G3BP1 fluorescence per SG for all SGs in an ROI. Exposure to smoke extract for 2 h resulted in the formation of largest and most abundant SGs. (**A**) Cluster analysis showed that the SG number per ROI was equal at 1–4 h of smoke extract exposure and significantly decreased at 6 h, suggesting disassembly. (#SG_1h_ = 82 ± 3, #SG_2h_ = 85 ± 3, #SG_4h_ = 71 ± 4, #SG_6h_ = 54 ± 2; N_1h_ = 4, n_1h_ = 40; N_2h_ = 4, n_2h_ = 40; N_4h_ = 3, n_4h_ = 30; N_6h_ = 4, n_6h_ = 40). (**B**) Cluster analysis further showed that SGs increased gradually in size over time, achieving a maximum size of 1.34 µm^2^ at 2 h post-smoke-extract exposure which significantly decreased at 6 h. (Size_1h_ = 1.09 ± 0.03, Size_2h_ = 1.34 ± 0.04, Size_4h_ = 1.19 ± 0.06, Size_6h_ = 1.12 ± 0.05 µm^2^; N_1h_ = 4, n_1h_ = 40; N_2h_ = 4, n_2h_ = 40; N_4h_ = 3, n_4h_ = 30; N_6h_ = 4, n_6h_ = 40.) (**C**) Mean G3BP1 fluorescence per SG was highest at 2 h smoke exposure and decreased significantly at shorter and longer exposure times suggesting incomplete assembly (at 1 h) and gradual disassembly (4 and 6 h), (N_1h_ = 4, n_1h_ = 40; N_2h_ = 4, n_2h_ = 40; N_4h_ = 3, n_4h_ = 30; N_6h_ = 4, n_6h_ = 39 (1 outlier was identified and excluded)). (**D**) The cumulative G3BP1 fluorescence per SG reached its maximum at 2 h smoke exposure and decreased significantly at 1, 4 and 6 h (N_1h_ = 4, n_1h_ = 40; N_2h_ = 4, n_2h_ = 40; N_4h_ = 3, n_4h_ = 30; N_6h_ = 4, n_6h_ = 40). (**E**) The sum of cumulative G3BP1 fluorescence per SG in an ROI was highest at 2 h post-smoke exposure, suggesting an optimal SG formation. This measurement gradually and significantly decreased at 4 and 6 h again, suggesting disassembly (N_1h_ = 4, n_1h_ = 39 (1 outlier is identified and excluded); N_2h_ = 4, n_2h_ = 40; N_4h_ = 3, n_4h_ = 29 (1 outlier is identified and excluded); N_6h_ = 4, n_6h_ = 40). (**F**) According to nucleus-based image analysis, cytoplasmic G3BP1 was most abundant at 2 and 4 h post-smoke-extract exposure and significantly decreased at 1 and 6 h (N_1h_ = 4, n_1h_ = 40; N_2h_ = 4, n_2h_ = 40; N_4h_ = 3, n_4h_ = 30; N_6h_ = 4, n_6h_ = 40). The nonparametric Kruskal–Wallis test was used to calculate significance with *: *p* < 0.02, **: *p* < 0.002, ***: *p* < 0.0006 and ****: *p* < 0.0001. All other comparisons are not significantly different. Each ROI is an independent biological sample. Data are presented as mean ± SEM. Abbreviations: SEM = standard error of mean, SG = stress granule, ROI = region of interest, N = total number of independent immunofluorescence experiments, n = total number of technical replicates (or analyzed ROIs) in all immunofluorescence experiments (n = N × number of technical replicates per experiment) and #SG = total number of SGs per ROI.

We then characterized SG formation in response to 15% smoke concentration at various exposure times (1, 2, 4 and 6 h) as shown in Figure 3E–H. According to visual inspection and cluster image analysis, SGs matured in both number (Figure 3F and Figure 5A) and size (Figure 5B) at 2 h post-smoke exposure. SG number and size slowly decreased at 4 and 6 h post-exposure, suggesting gradual disassembly (Figure 3G,H and Figure 5A,B). The mean G3BP1 fluorescence level per SG (Figure 5C), the cumulative G3BP1 fluorescence level per SG (Figure 5D) and per ROI (Figure 5E) were highest at 2 h post-smoke-extract exposure and gradually decreased at longer exposure times. Our findings suggest that SG formation is complete at 2 h post-smoke-extract exposure, and SGs undergo gradual disassembly at longer exposure times.

**Figure 6:**
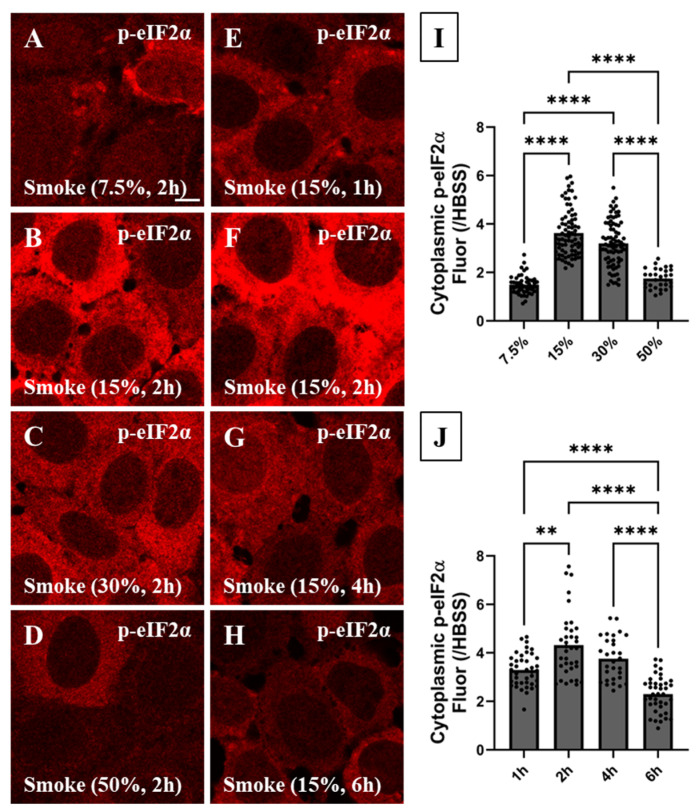
Characterization of eIF2α phosphorylation in response to different smoke extract concentrations and times. CFBE cells were exposed to different smoke extract concentrations (7.5, 15, 30, 50%) for different exposure times (1, 2, 4, 6 h) as shown in Figure 2. The aim of this experiment is to correlate SG formation in Figure 2 to phosphorylated eIF2α (p-eIF2α, red) level using IF imaging and cluster image analysis under the above-stated conditions. (**A**–**D**) The highest increase in p-eIF2α level was observed under 15% smoke extract exposure (**B**) under which condition SGs were most mature in Figure 2. p-eIF2α level decreased gradually under 30 and 50% smoke extract concentrations. (**E**–**H**) The highest increase in p-eIF2α level was observed under 2 h exposure time (**F**) under which condition SG formation was maximal in Figure 2. The p-eIF2α level decreased gradually at 4 and 6 h exposure times. (**I**) The mean cytoplasmic p-eIF2α fluorescence under 7.5, 15, 30, and 50% smoke extract concentrations was measured using the nucleus-based image analysis and normalized to its corresponding HBSS treatment. The cytoplasmic p-eIF2α level was highest under 15% smoke extract exposure and decreased gradually under 30 and 50% smoke concentrations (N_7.5%_ = 5, n_7.5%_ = 46 (4 outliers were identified and excluded); N_15%_ = 8, n_15%_ = 80; N_30%_ = 8, n_30%_ = 78; N_50%_ = 3, n_50%_ = 30). (**J**) The mean cytoplasmic p-eIF2α fluorescence was also measured using the nucleus-based image analysis under 15% smoke extract for 1, 2, 4, and 6 h. The mean cytoplasmic p-eIF2α level was highest under 2 h exposure time and decreased gradually at 4 and 6 h, suggesting dephosphorylation (N_1h_ = 4, n_1h_ = 40; N_2h_ = 4, n_2h_ = 36 (4 outliers were identified and excluded); N_4h_ = 3, n_4h_ = 30; N_6h_ = 4, n_6h_ = 40). Confocal image scale bar = 7.7 µm. The nonparametric Kruskal–Wallis test was used to calculate significance with **: *p* < 0.0022 and ****: *p* < 0.0001. All other comparisons are not significantly different. Each ROI is an independent biological sample. Data are presented as the mean ± SEM. Abbreviations: SEM = standard error of mean, ROI = region of interest, SG = stress granule, N = total number of independent immunofluorescence experiments, and n = total number of technical replicates (or analyzed ROIs) in all immunofluorescence experiments (n = N × number of technical replicates per experiment).

A quantitative nucleus-based image analysis was then used to measure the cytoplasmic G3BP1 level. This analysis takes advantage of nuclear DAPI signal to segregate nuclear and cytoplasmic fluorescence of a protein of interest in an ROI (see Figure S1A–H for corresponding DAPI signal of cells in Figure 3A–H and Figure 6A–H). Using this analysis, we measured the cumulative cytoplasmic G3BP1 fluorescence level because the protein is not expressed in the nucleus. Cumulative cytoplasmic G3BP1 fluorescence was highest when cells were exposed to 15% smoke for 2 h (Figure 4F and Figure 5F). To support our quantitative IF imaging findings, immunoblotting showed that cellular G3BP1 abundance significantly increased by ~4-fold in response to smoke extract (Figure 3I,J). Taken together, SG formation maturation and the maximal increase in G3BP1 expression and sequestration into SGs occurred under 15% smoke extract exposure for 2 h.

### 3.3. SG Formation in Response to Smoke Exposure Is Canonical

Next, we hypothesized that SGs in smoke-treated cells form canonically by activating the ISR pathway through eIF2α phosphorylation. To test this hypothesis, the cellular distribution and abundance of phosphorylated eIF2α (p-eIF2α) in CFBE cells was assessed using IF imaging. Compared to cells exposed to a vehicle control (Figure 1C), IF imaging demonstrated a marked increase in p-eIF2α level in cell cytoplasm in response to 15% smoke exposure for 2 h (Figure 1D), validating our hypothesis. SG formation in primary human bronchial epithelial cells was also associated with a significant 10-fold increase in p-eIF2α level, as shown using immunoblotting (Figure 2E,F), demonstrating canonical SG formation.

Cytoplasmic p-eIF2α abundance under various smoke concentrations and exposure times was measured in CFBE cells using IF imaging and the nucleus-based image analysis. When cells were exposed to increasing smoke concentrations (Figure 6A–D), visual inspection and nucleus-based image analysis showed that cytoplasmic p-eIF2α level was highest under 15% smoke extract exposure (Figure 6B,I, 4-fold). Higher smoke extract concentrations (30 and 50%) resulted in significantly lower p-eIF2α abundance (Figure 6C,D,I) which is associated with and potentially explains the significant decrease in SG number and size under these exposure conditions. When cells were exposed to increasing smoke exposure times (Figure 6E–H), the cytoplasmic p-eIF2α level was highest under 2 h smoke extract exposure (Figure 6F,J, 4-fold). SG disassembly at longer exposure times (4 and 6 h) was also associated with reduced p-eIF2α providing a molecular cause. Based on these results, we conclude that SG characteristics are tightly regulated by p-eIF2α level. Taken together, SG formation maturation and maximal ISR activation as measured by the increase in cytoplasmic p-eIF2α level occurred under 15% smoke extract exposure for 2 h. Therefore, this condition was used for the rest of the study to investigate the molecular origins of SG formation in response to acute smoke exposure.

**Figure 7:**
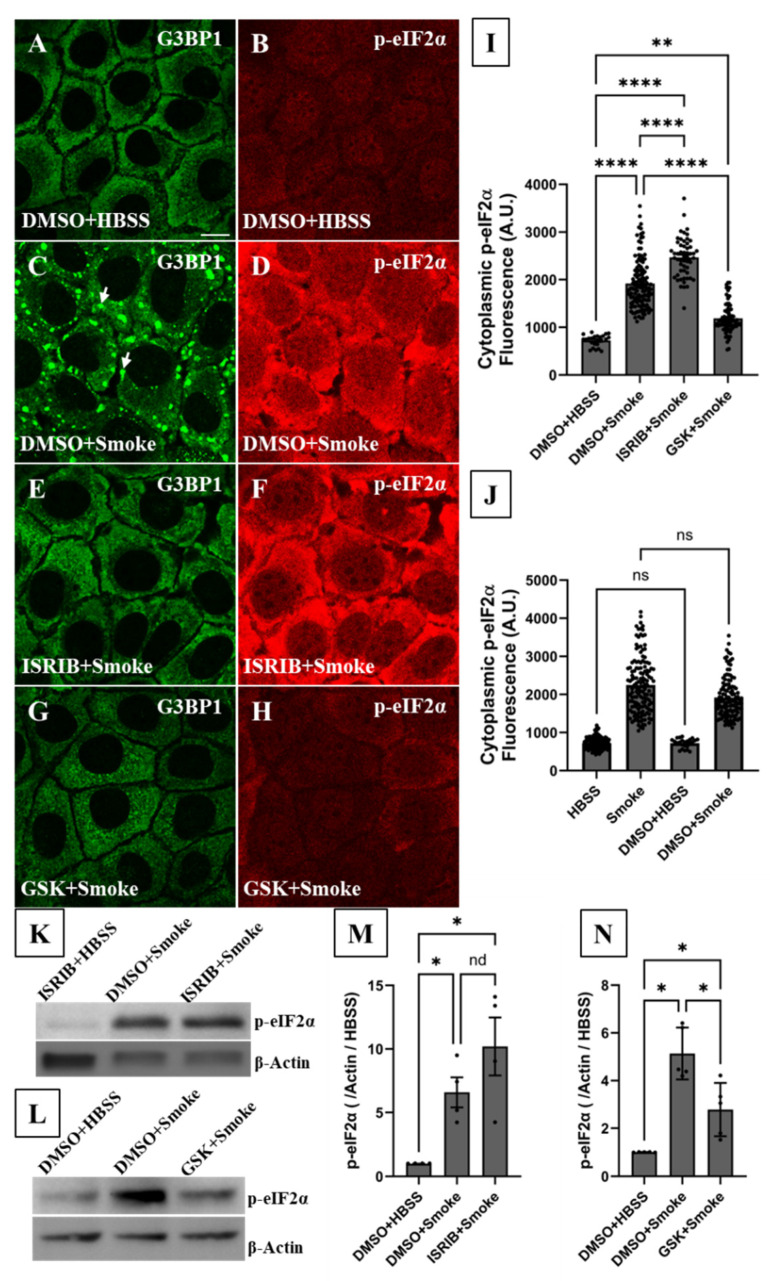
SG formation is dependent on the PERK/eIF2α signaling pathway. CFBE cells were pretreated with DMSO, ISRIB or GSK and then exposed to 15% HBSS or smoke extract for 2 h. IF imaging or immunoblotting were then performed. (**A**,**B**) IF imaging showed that endogenous G3BP1 (**A**) and p-eIF2α (**B**) are homogenously distributed in the cell cytoplasm under control conditions (DMSO + HBSS). (**C**) Smoke exposure induced robust SG formation (white arrows) and (**D**) a significant increase in cytoplasmic eIF2α phosphorylation (p-eIF2α). (**E**,**F**) Pretreating cells with ISRIB, an inhibitor of p-eIF2α function, at 200 nM for 1 h prior to smoke extract exposure (**E**) completely abolished SG formation, establishing causality (**F**) without reducing p-eIF2α abundance. (**G**,**H**) Inhibiting p-PERK by pretreating cells with 2 µM GSK for 1 h prior to smoke extract exposure (**G**) completely blocked SG formation and (**H**) abrogated the increase in p-eIF2α levels, indicating that SG formation is PERK/eIF2α-dependent. (**I**) Quantitative nucleus-based image analysis of mean cytoplasmic p-eIF2α levels demonstrated a significant increase in cells’ response to smoke extract exposure as demonstrated by the 3-fold increase in cytoplasmic p-eIF2α fluorescence intensity (IF_DMSO+HBSS_ = 724 ± 25, N_DMSO+HBSS_ = 4, n_DMSO+HBSS_ = 24; IF_DMSO+Smoke_ = 1919 ± 48, N_DMSO+Smoke_ = 16, n_DMSO+Smoke_ = 120 (4 outliers were identified and excluded)). The analysis also showed that ISRIB pretreatment induced further accumulation and a significant increase in cytoplasmic p-eIF2α levels in response to smoke exposure (IF_ISRIB+Smoke_ = 2464 ± 57, N_ISRIB+Smoke_ = 7, n_ISRIB+Smoke_ = 54). GSK pretreatment, on the other hand, significantly attenuated p-eIF2α increase in response to smoke exposure (IF_GSK+Smoke_ = 1187 ± 34, N_GSK+Smoke_ = 11, n_GSK+Smoke_ = 82) without fully returning to the DMSO + HBSS baseline. (**J**) Quantitative nucleus-based image analysis of mean cytoplasmic p-eIF2α levels showed that pretreating cells with DMSO (drug vehicle control) did not induce significant change in cytoplasmic p-eIF2α levels under HBSS or smoke exposure conditions (IF_HBSS_ = 725 ± 13, N_HBSS_ = 18, n_HBSS_ = 132 (1 outlier was identified and excluded); IF_Smoke_ = 2249 ± 62, N_Smoke_ = 19, n_Smoke_ = 142). The nonparametric Kruskal–Wallis test was used to calculate significance in (**I**,**J**). ns: not significant, **: *p* = 0.01 and ****: *p* < 0.0001. Each ROI is an independent biological sample. (**K**–**N**) immunoblotting demonstrated a significant 5- to 7-fold increase in cellular p-eIF2α level in response to smoke exposure, with ISRIB pretreatment increasing this level non-significantly to 10-fold (N = 4, IB_DMSO+HBSS_ = 1, IB_DMSO+Smoke_ = 7 ± 1, IB_ISRIB+Smoke_ = 10 ± 2). GSK pretreatment reduced p-eIF2α levels significantly but not to the DMSO + HBSS basal level (N = 5, IB_DMSO+HBSS_ = 1, IB_DMSO+Smoke_ = 5.1 ± 0.5, IB_GSK+Smoke_ = 2.8 ± 0.5). Immunoblotting data were normalized to β-Actin protein level and then to the corresponding vehicle control. See also Figures S4 and S5. Brown–Forsythe and Welch ANOVA tests were used to calculate significance in (**M**,**N**), with nd = no discovery and *: *p* < 0.03. Data are presented as mean ± SEM. Confocal image scale bar = 12.8 µm. Abbreviations: SEM = standard error of mean, SG = stress granule, ROI = region of interest, IF = immunofluorescence, IB = immunoblotting, N = the total number of independent immunofluorescence experiments and n = total number of technical replicates (or analyzed ROIs) in all immunofluorescence experiments (n = N × number of technical replicates per experiment). Western blot original images can be found in Supplementary Materials.

To determine whether there is a causal relationship between p-eIF2α increase and SG formation, p-eIF2α function was pharmacologically inhibited by pretreating CFBE cells with ISRIB (200 nM, 1 h), which blocks the downstream functional effects of p-eIF2α without reducing its abundance [26,41,42,43]. According to IF imaging and compared to smoke exposure alone (Figure 7C), ISRIB completely blocked SG formation in response to smoke (Figure 7E), supporting a causal relationship and canonical formation. ISRIB pretreatment also significantly increased cytoplasmic p-eIF2α abundance in smoke-treated cells as shown by quantitative IF imaging (Figure 7F,I). Cells treated with DMSO (ISRIB’s vehicle control) behaved like those exposed to no vehicle control (Figure 7J), indicating that DMSO does exert off-target effects. Immunoblotting fully supported IF imaging results and demonstrated 5- to 7-fold increase in cellular p-eIF2α in response to smoke exposure (Figure 7K–N). Immunoblotting also measured a further increase in cellular p-eIF2α abundance (by 10-fold) when cells were pretreated with ISRIB prior to smoke extract exposure (Figure 7K,M), suggesting accumulation upon inhibiting its downstream function.

## 4. PERK Activates the Integrated Stress Response Pathway in Response to Smoke Exposure

**Figure 8:**
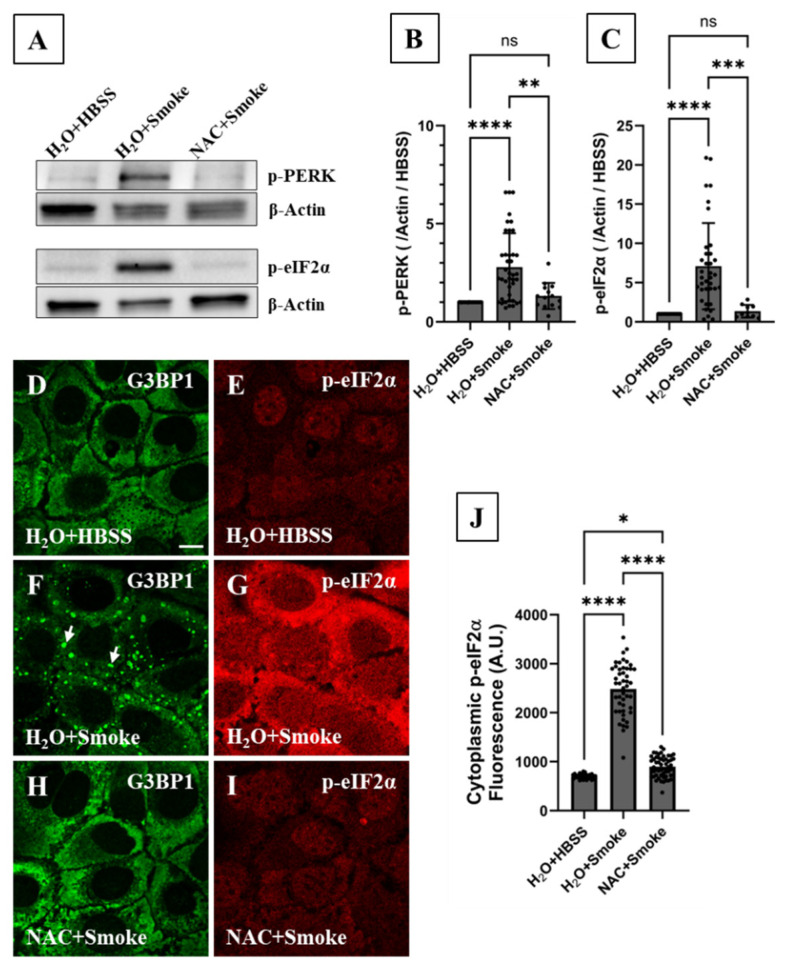
Reactive oxygen species activate the PERK/eIF2α signaling pathway and induce SG formation in response to smoke exposure. CFBE cells were pretreated with H_2_O or 5 mM NAC for 15 min and then exposed to 15% HBSS or smoke extract for 2 h. IF imaging and immunoblotting analyses were then performed. (**A**–**C**) Immunoblotting (IB) demonstrated a significant 7-fold increase in cellular p-eIF2α level in response to smoke exposure which was fully abolished upon pretreating cells with NAC (IB_H2O+HBSS_ = 1, IB_H2O+Smoke_ = 7.1 ± 0.9, IB_NAC+Smoke_ = 1.4 ± 0.3; N_H2O+HBSS_ = N_H2O+Smoke_ = 36, N_NAC+Smoke_ = 10). Additionally, immunoblotting showed that smoke exposure increased p-PERK level by 2.8-fold, which is also completely abolished by NAC pretreatment (IB_H2O+HBSS_ = 1, IB_H2O+Smoke_ = 2.8 ± 0.3, IB_NAC+Smoke_ = 1.3 ± 0.2; N_H2O+HBSS_ = 41, N_H2O+Smoke_ = 40, N_NAC+Smoke_ = 14). IB data were normalized to β-Actin protein level and then to the corresponding vehicle control. See also Figures S6 and S7. (**D**,**E**) Confocal IF imaging showed that (**D**) endogenous G3BP1 and (**E**) p-eIF2α were homogenously distributed in the cell cytoplasm under control conditions (H_2_O + HBSS). (**F**,**G**) IF imaging showed that smoke extract exposure induced SG formation (white arrows) and a marked increase in cytoplasmic p-eIF2α abundance. (**H**,**I**) Pretreating cells with the ROS scavenger NAC prior to smoke extract exposure completely abolished SG formation and inhibited the increase in p-eIF2α levels, establishing that SG formation is ROS-driven in p-eIF2α-dependent manner. (**J**) Quantitative nucleus-based image analysis showed that NAC pretreatment significantly attenuated the significant 3-fold increase in cytoplasmic p-eIF2α level in response to smoke extract exposure (IF_H2O+HBSS_ = 700 ± 9, IF_H2O+Smoke_ = 2485 ± 75, IF_NAC+Smoke_ = 899 ± 28; N_H2O+HBSS_ = 5, n_H2O+HBSS_ = 30; N_H2O+Smoke_ = 8, n_H2O+Smoke_ = 47 (1 outlier was identified and excluded); N_NAC+Smoke_ = 9, n_NAC+Smoke_ = 54). Each ROI is an independent biological sample. The nonparametric Kruskal–Wallis test was used to calculate significance in (**B**,**C**,**J**). ns: not significant, *: *p* < 0.03, **: *p* = 0.0095, ***: *p* = 0.0002, and ****: *p* < 0.0001. Data are presented as mean ± SEM. Confocal image scale bar = 12.8 µm. Abbreviations: SEM = standard error of mean, SG = stress granule, ROI for region of interest, IF = immunofluorescence, IB = immunoblotting, N = the total number of independent immunofluorescence experiments, and n = the total number of technical replicates (or analyzed ROIs) in all immunofluorescence experiments (n = N × number of technical replicates per experiment). Western blot original images can be found in Supplementary Materials.

Since oxidative stress induces PKR phosphorylation, and knowing smoke contains a high concentration of reactive oxygen species (ROS) [8,30], we initially hypothesized that SG formation and eIF2α phosphorylation are driven by PKR phosphorylation (p-PKR) [25,26,27,32]. To examine this hypothesis, three different p-PKR antibodies were used; two targeted the Thr446 phosphorylation site and one targeted both Thr446 and Thr451 phosphorylation sites. According to immunoblotting results, no increase in p-PKR abundance beyond the basal level was observed in smoke-treated cells (Figure S8). Furthermore, pretreating cells with the specific PKR inhibitor C16 at 1 µM for 1 h prior to smoke exposure did not reduce p-eIF2α abundance (Figure S8). Our results suggest that PKR does not play a role in activating the ISR under acute smoke exposure conditions. Since ROS induce endoplasmic reticulum (ER) stress, we then hypothesized that PERK [25,26] phosphorylation (p-PERK) takes place and is responsible for SG formation by increasing p-eIF2α abundance following smoke exposure. Immunoblotting showed that smoke exposure significantly increased p-PERK abundance by 2.8-fold (Figure 8A,B), which was associated with a significant increase in p-eIF2α by 7-fold (Figure 8A,C). Similarly, p-PERK abundance in primary human bronchial epithelial cells demonstrated a significant 2-fold increase in response to smoke exposure (Figure 2E,G).

To determine whether there is a causal relationship between PERK phosphorylation and both p-eIF2α elevation and SG formation under smoke exposure conditions, p-PERK function was pharmacologically inhibited by pretreating CFBE cells with GSK2606414 [44] (GSK, 2 µM, 1 h), a p-PERK specific inhibitor. SG formation was visualized by IF imaging while p-eIF2α abundance was measured by the quantitative nucleus-based image analysis and immunoblotting. According to IF imaging, GSK pretreatment abolished SG formation in smoke-treated cells (Figure 7G). GSK also significantly decreased p-eIF2α levels as shown by IF imaging (Figure 7H,I) and immunoblotting (Figure 7L,N). Our results support a causal relationship and clearly demonstrate a role for PERK/eIF2α signaling in human bronchial epithelial cells’ response to smoke.

## 5. Reactive Oxygen Species Activate the ISR and Induce SG Formation in Response to Smoke Exposure

Smoke contains a high concentration of reactive oxygen species (ROS) [30] and hydrogen peroxide has been shown to induce SG formation [45]. Thus, we hypothesized that smoke-associated ROS trigger SG formation in a PERK/eIF2α-dependent manner under our experimental conditions. Treating cells with the ROS scavenger N-acetylcysteine (NAC, 5 mM) 15 min before smoke extract exposure completely abolished SG formation (Figure 8H) and inhibited the increase in p-eIF2α and p-PERK abundance as measured by immunoblotting (Figure 8A–C) and IF imaging (Figure 8I,J). The ability of NAC to inhibit SG formation and abrogate the increase in p-eIF2α and p-PERK levels following smoke exposure reveals that smoke-associated ROS are the driving force. Our results clearly show that canonical SG formation in response to smoke in human bronchial epithelial cells is ROS-driven and that ROS action is upstream of PERK.

## 6. Discussion

This study uses quantitative fluorescence imaging analyses and immunoblotting, two essential cell biology tools, to investigate the response of human bronchial epithelial cells to acute smoke extract exposure. While chronic (24 h) exposure to smoke extract triggers inflammasome formation leading to cytokines maturation and secretion [15,16,17,19], we show here that acute smoke exposure triggers canonical SG formation through activating the protective ISR pathway via phosphorylating the PERK/eIF2α arm in ROS-dependent manner. We also characterize SG formation and associated p-eIF2α abundance in response to different smoke extract concentrations and exposure times and report that optimal SG assembly, sequestration of G3BP1 and cytoplasmic p-eIF2α accumulation occur at 2 h post-exposure to 15% smoke extract. This is followed by gradual disassembly at longer exposure times such as 4 and 6 h post-exposure.

**Relevance to smoking:** Smoke was bubbled in HBSS immediately before cell exposure, contained the gas and tar (particulate matter) phases, and was not filtered for maximal potency. HBSS mimics biological fluids, making it similar to human airway surface liquid, and thus a suitable vehicle with reduced confounding effects. For IF imaging, approximately 1.5 × 10^5^ cells are exposed to ~150 µL of 100% smoke extract for a final 15% smoke extract concentration. This means every cell is exposed to ~10^−3^ µL of 100% smoke extract. Comparatively, smoking 1 cigarette is equivalent to distributing 10 mL of 100% smoke extract over the entire airway epithelium. This translates into the experimental parallel of 1 cigarette bubbled in 10 mL of HBSS. Based on personal experience related to isolation of human airway epithelial cells from donors’ airways, an adult airway epithelium is composed of ~50 million surface epithelial cells. This means that every airway epithelial cell is exposed to ~2 × 10^−4^ µL of 100% smoke extract. A simple calculation reveals that under our experimental conditions, every CFBE cell is exposed to ~5× more smoke extract than a lung epithelial cell is upon smoking 1 cigarette. This demonstrates that our experimental conditions represent a heavy smoking event and not mere secondhand smoking exposure.

**PERK or PKR:** Although PKR phosphorylation was anticipated in response to oxidative stress as previously reported [25,26,27,32], no increase in PKR phosphorylation was detected using immunoblotting under our smoke extract exposure conditions. Additionally, PKR inhibition has no effect on p-eIF2α abundance in response to smoke exposure, again indicating that PKR does not induce the ISR mechanism in this study. Instead, our results demonstrate a significant increase in p-PERK abundance causing p-eIF2α elevation and SG formation. Inhibiting PERK significantly decreases p-eIF2α. This strongly suggests that smoke-induced oxidative stress induces ER stress leading to PERK phosphorylation and ISR activation. Previously, Tagawa et al. reported that cigarette smoke extract prepared from the solid (tar) phase increases CHOP expression, resulting in apoptosis in ROS-dependent manner in the human bronchial epithelial cell line BEAS-2B [29]. The authors show that this increase is due to the induction of the unfolded protein response (UPR) pathway via activating the PERK/eIF2α branch. Despite the differences in smoke preparation and cell lines used, both Tagawa et al. and this study report PERK/eIF2α phosphorylation. It is worth emphasizing that the two studies complement each other by focusing on different outcomes of the ISR mechanism. Tagawa et al. reported PERK/eIF2α phosphorylation at 1–6 h post-extract-exposure with a focus on the induction of both ATF4/CHOP expression and apoptosis, as well as the responsible reactive oxygen species [29]. Our study, on the other hand, investigates canonical SG formation, which was not previously reported, and meticulously characterizes this process under different smoke exposure conditions. We also reveal that SG characteristics are closely regulated by p-eIF2α level, with lower levels resulting in smaller and fewer SGs. Contrary to what was shown in Tagawa et al. [29], we did not observe cell rounding (apoptosis) under smoke extract exposure for up to 6 h, which could be related to differences in smoke extract preparation or cell types. While the previous study [29] uses only the tar phase, which is dissolved in PBS and stored at −80 °C until use, our study uses freshly prepared extract that contains both the gas and tar phases to more closely mimic cigarette smoking. The extract concentration calculations in both studies are also different, potentially resulting in differences in smoke extract potency.

**Characterizing SG maturation:** To identify the mechanism responsible for SG formation, we began by characterizing their formation under different smoke extract concentrations ranging from 7.5% to 50%. This characterization relies on quantitative IF imaging to measure formation parameters such as SG size and count, and the amount of sequestered G3BP1 therein per SG and an ROI. G3BP1 is essential for SG formation and thus is used here as an SG marker to characterize the formation process. G3BP1 distribution and fluorescence level allowed for the retrieval of these parameters using cluster analysis. This analysis demonstrated that SGs optimally assemble when cells are exposed to 15% smoke extract. Under this exposure condition, SGs were largest, most abundant and sequestered the most G3BP1. This condition was also associated with the highest cytoplasmic G3BP1 and p-eIF2α abundance. Exposure to higher smoke extract concentrations, such as 30% and 50%, resulted in the formation of smaller, less abundant SGs with less G3BP1 content. Reductions in cytoplasmic G3BP1 and p-eIF2α also occurred under these concentrations. Taken together, our results indicate that the assembly process is dictated by eIF2α phosphorylation. The reason behind the reduction in cytoplasmic p-eIF2α under excessive oxidative stress condition is under investigation.

We then characterized SG formation under 15% smoke extract concentration and exposure times ranging from 1 to 6 h. The choice of 6 h is based on an observation that smoke-induced SGs do not disassemble as fast as those induced by sodium arsenite (3–4 h) [46,47]. This difference is likely due to a difference in the cell type and/or initiating stress. Although large SGs formed at as early as 1 h post-exposure, equally large but more abundant SGs formed at 2 h post-exposure. G3BP1 sequestration and cytoplasmic G3BP1 and p-eIF2α abundance were also maximal at 2 h post-exposure. Disassembly is evident at longer exposure times such as 4 and 6 h. We conclude that exposure to 15% smoke extract for 2 h is sufficient to induce maximal SG formation due to maximal increase in G3BP1 expression and eIF2α phosphorylation. Therefore, this condition was used to further investigate the molecular causes of SG formation.

**Reconciling quantitative IF imaging and immunoblotting results:** In this study, SG formation in response to smoke extract exposure is canonical and caused by a significant increase in p-eIF2α abundance. According to quantitative nucleus-based image analysis, smoke exposure increased cytoplasmic p-eIF2α level by 3.5-fold while immunoblotting demonstrated 5- to 7-fold increase in cellular p-eIF2α. This mild difference can be reconciled by understanding a fundamental difference in both methods. Since IF imaging showed low nuclear p-eIF2α abundance and negligible increase therein in response to smoke exposure, we assume that the increase in cellular p-eIF2α is proportional to an increase in its cytoplasmic abundance. We propose that the difference in fold-increase in quantitative IF imaging and immunoblotting are explained by contributions of cell autofluorescence and nonspecific binding of the primary and secondary antibodies in quantitative IF imaging. Based on immunoblotting, we found that nonspecific binding of p-eIF2α antibody is very negligible. Based on IF imaging, mean cytoplasmic autofluorescence in the absence of primary and secondary antibodies is ~35% of the mean cytoplasmic p-eIF2α fluorescence signal under HBSS exposure condition (15% for 2 h, Figure S9B). In the presence of a fluorescent secondary antibody, the mean cytoplasmic autofluorescence combined with the contribution of the secondary antibody nonspecific binding is ~45% of mean cytoplasmic p-eIF2α fluorescence signal under HBSS exposure condition. Exposure to smoke did not increase the level of background fluorescence. Knowing that 45% of the mean cytoplasmic p-eIF2α fluorescence signal measured under HBSS treatment condition is related to both autofluorescence and nonspecific binding and that smoke does not change this contribution, subtracting this amount equally from the fluorescence signal under HBSS and smoke extract exposure conditions corrects the relative increase in mean p-eIF2α fluorescence level under smoke exposure condition to over 6-fold matching measured by immunoblotting. This also suggests that IF imaging is an alternative method to immunoblotting for measuring protein levels, provided that the primary antibody is highly specific and that control experiments to evaluate the relative background fluorescence level are performed.

## 7. Conclusions

We show for the first time that acute exposure to cigarette smoke extract triggers canonical SG formation in human bronchial epithelial cells by activating the PERK/eIF2α pathway in ROS-dependent manner. We further show that SG formation in terms of size, abundance and sequestration of G3BP1 is dependent on smoke extract concentration and duration. High smoke concentrations reduce p-eIF2α elevation, which disrupts SG formation, resulting in smaller and fewer SGs. Our results also show that smoke-induced SGs fully mature at 2 h post 15% smoke extract exposure and then undergo gradual disassembly. Our results demonstrate that SG maturation is associated with a significant increase in cellular G3BP1 expression, suggesting that its mRNA escapes protein translation inhibition expected from SG formation. We propose that acute exposure to cigarette smoke activates the protective integrated stress response mechanism in human bronchial epithelial cells to increase adaptation to stress.

## Data Availability

This study did not generate new unique reagents. The original contributions presented in this study are included in the article/Supplementary Material. Further inquiries can be directed to the corresponding author.

## Supplementary Materials

The following supporting information can be downloaded at https://www.mdpi.com/article/10.3390/biom16040615/s1, The supplementary document contains Supplementary Figures S1–S9. Figure S1: Nuclear DAPI staining for CFBE cells exposed to increasing smoke extract concentrations and exposure times. Figure S2: Stress granule formation in primary human bronchial epithelial cells is canonical. Figure S3: Smoke extract exposure increases G3BP1 abundance in CFBE cells. Figure S4: Inhibiting p-eIF2α function using ISRIB increases smoke-induced p-eIF2α abundance in CFBE cells. Figure S5: Inhibiting p-PERK using GSK attenuates smoke-induced p-eIF2α abundance in CFBE cells. Figure S6: Reactive oxygen species induce smoke-associated PERK phosphorylation in CFBE cells. Figure S7: Reactive oxygen species induce smoke-associated eIF2α phosphorylation in CFBE cells. Figure S8: Smoke extract exposure does not induce PKR phosphorylation in CFBE cells. Figure S9: Quantification of the contribution of mean cytoplasmic autofluorescence and non-specific binding to mean cytoplasmic G3BP1 and p-eIF2α fluorescence intensity.
