# Supplementary material for: Cigarette Smoke Induces Canonical Stress Granule Formation in Human Bronchial Epithelial Cells in Reactive Oxygen Species- and PERK-Dependent Manners
Source: Biomolecules. 2026 Apr 21;16(4):615. doi: 10.3390/biom16040615 (PMC13113878; doi:10.3390/biom16040615)
Supplement: Supplementary file 1 [file biomolecules-16-00615-s001.zip › biomolecules-4200432-supplementary.pdf]

Supplementary Material for

Cigarette Smoke Induces Canonical Stress Granule Formation in Human Bronchial Epithelial Cells in Reactive Oxygen Species- and PERK-Dependent Manners

Mousumi Bhowmik [1,†], Chenkun Zheng [1,†], Bisrat Bekele [1], Jessica Failler [1], Carlie Klatt [2], Souren Farimani [3], Bryant Jones [4], Chung-Chun Tyan [5] and Asmahan Abu-Arish [1,*]

[1] Department of Anatomy, Physiology and Pharmacology, University of Saskatchewan, Saskatoon, SK S7N 5E5, Canada; ukc521@mail.usask.ca (M.B.); yiq173@mail.usask.ca (C.Z.); mzl610@mail.usask.ca (B.B.); jdp618@mail.usask.ca (J.F.)
[2] Regina General Hospital, Regina Campus, University of Saskatchewan, Regina, SK S4P 0W5, Canada; cak278@mail.usask.ca
[3] Department of Neurology and Neurosurgery, Montreal Neurological Institute, McGill University, Montreal, QC H3A 2B4, Canada; souren.vahdatfarimani@mail.mcgill.ca
[4] School of Rehabilitation Science, University of Saskatchewan, Saskatoon, SK S7N 2Z4, Canada; bwj823@mail.usask.ca
[5] Division of Respirology, Critical Care and Sleep Medicine, University of Saskatchewan, Saskatoon, SK S7N 0W8, Canada
* Correspondence: asmahan.abuarish@usask.ca
† These authors contributed equally to this work.

This file includes:

Figures S1–S9

Supplementary Information Figures

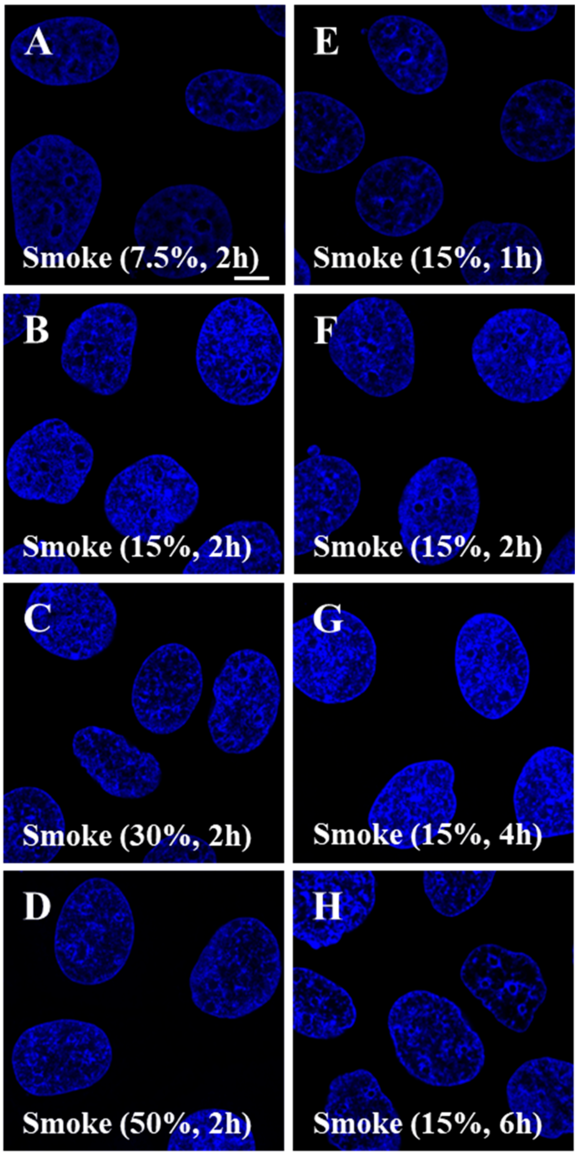

Figure S1: Nuclear DAPI staining for CFBE cells exposed to increasing smoke extract concentrations and exposure times. (A – H) Corresponding nuclear DAPI staining for CFBE cells shown in Figures 3A–H and 6A–H in the main manuscript. CFBE cells were exposed to increasing smoke extract concentrations for different exposure times. IF imaging was performed to visualize nuclear DAPI staining for (A – D) cells exposed to 7.5, 15, 30 or 50% smoke extract for 2h or (E – H) cells exposed to 15% smoke extract for 1, 2, 4 and 6h. IF imaging shows that cells nuclei are intact under all smoke exposure conditions. DAPI staining is used throughout this study to define the nuclear contribution by a nucleus-based image analysis. The analysis segregates the nuclear and cytoplasmic contributions of the fluorescence intensity of a protein of interest such as G3BP1 and p-eIF2α to investigate the origins of SG formation. Scale bar=7.7 μm.

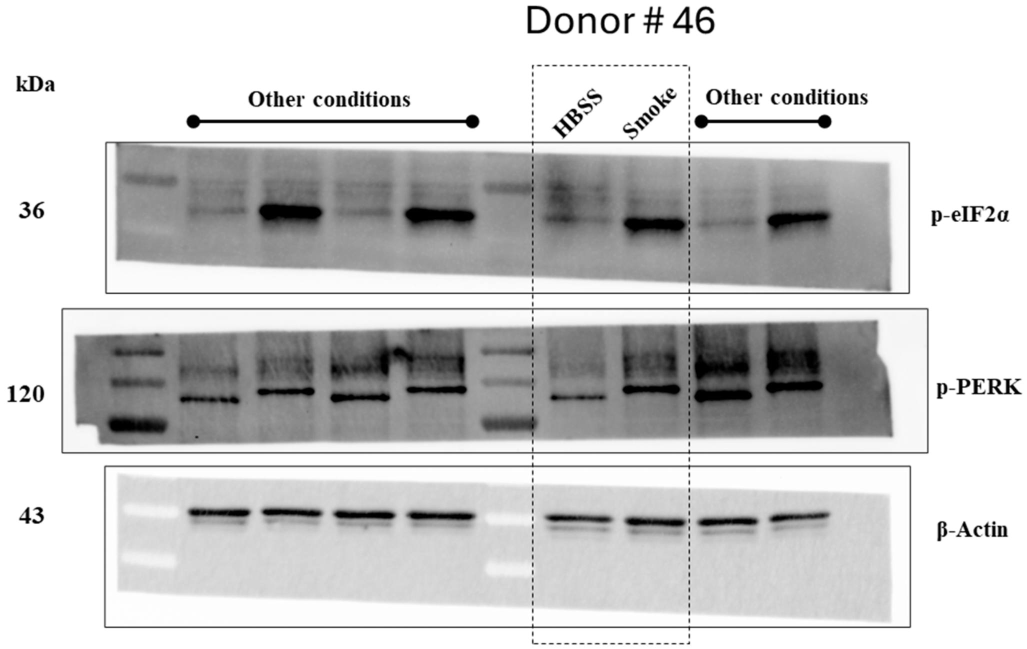

Figure S2: Stress granule formation in primary human bronchial epithelial cells is canonical. Smoke extract exposure markedly increased p-eIF2α abundance in primary human bronchial epithelial cells in p-PERK-dependent manner**.** Immunoblotting captures a significant 10-fold increase in p-eIF2α abundance and associated 2-fold increase in p-PERK abundance following exposure to 15% smoke extract for 2h (See Figure 2E–G in the manuscript).

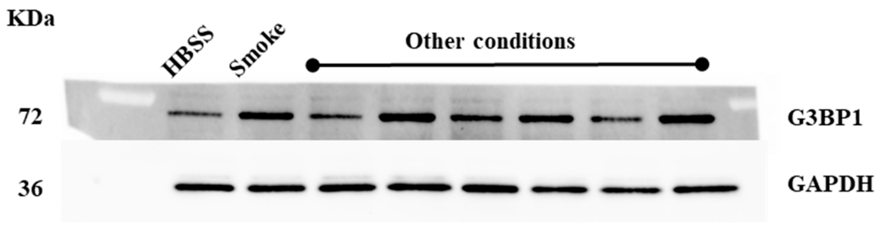

Figure S3: Smoke extract exposure increases G3BP1 abundance in CFBE cells. Immunoblotting captures a significant 4-fold increase in G3BP1 abundance following smoke extract exposure (See Figure 3I in the manuscript). This ratio is calculated by normalizing G3BP1 levels under smoke exposure conditions to both G3BP1 levels under HBSS exposure conditions and GAPDH levels.

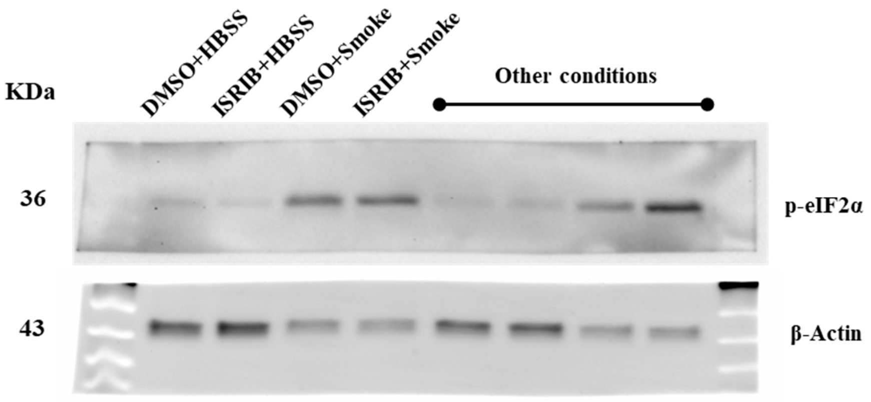

Figure S4: Inhibiting p-eIF2α function using ISRIB increases smoke-induced p-eIF2α abundance in CFBE cells. Immunoblotting captures a 7-fold increase in p-eIF2α abundance following smoke extract exposure (See Figure 7K,M in the manuscript). Pre-treatment cells with the p-eIF2α functional inhibitor ISRIB (200 nM, 1h) prior to smoke exposure completely abolishes SG formation without reducing p-eIF2α levels. Instead, an increase in p-eIF2α levels to 10-fold is measured. These ratios are calculated by normalizing to both p-eIF2α levels under ISRIB+HBSS exposure conditions and β-Actin levels.

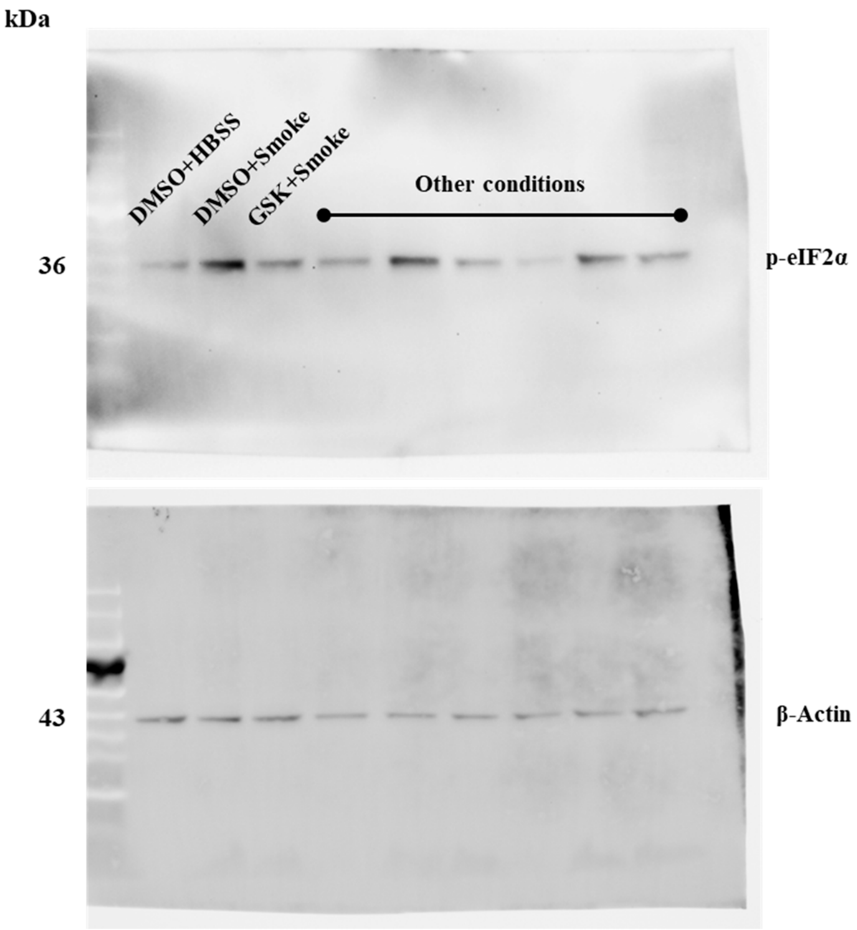

Figure S5: Inhibiting p-PERK using GSK attenuates smoke-induced p-eIF2α abundance in CFBE cells. Immunoblotting captures a 5-fold increase in p-eIF2α abundance following smoke extract exposure (See Figure 7L,N in the manuscript). Pre-treatment with the p-PERK inhibitor GSK (2 μM, 1h) prior to smoke exposure largely attenuates smoke-induced p-eIF2α increase. These ratios are calculated by normalizing to both p-eIF2α levels under DMSO+HBSS exposure conditions and β-Actin levels.

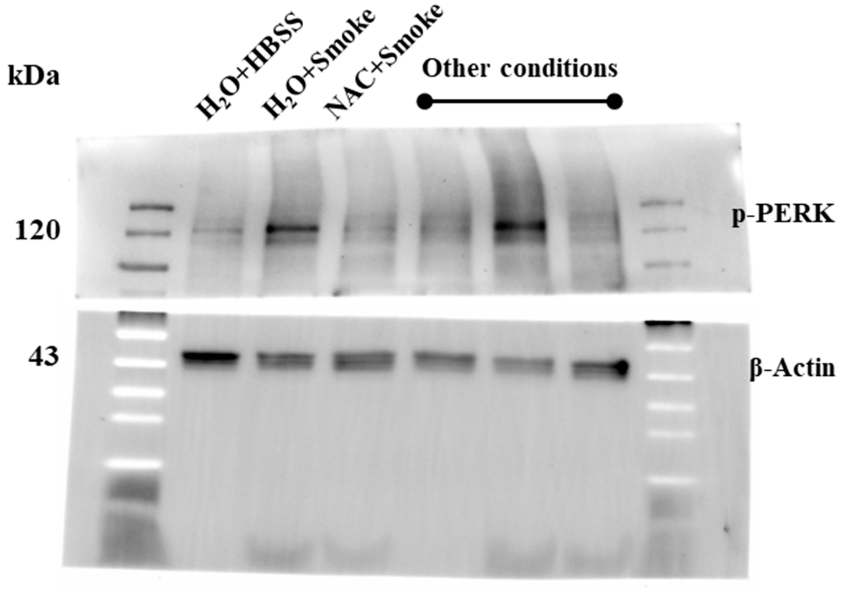

Figure S6: Reactive oxygen species induce smoke-associated PERK phosphorylation in CFBE cells. Immunoblotting captures a 2.8-fold increase in p-PERK abundance following smoke extract exposure (See Figure 8A,B in manuscript). Pre-treatment with the reactive oxygen species scavenger N-acetylcysteine (NAC, 5 mM, 15 min) prior to smoke exposure fully abrogates smoke-induced p-PERK increase. These ratios are calculated by normalizing to both p-PERK levels under H_2O+HBSS exposure conditions and β-Actin levels.

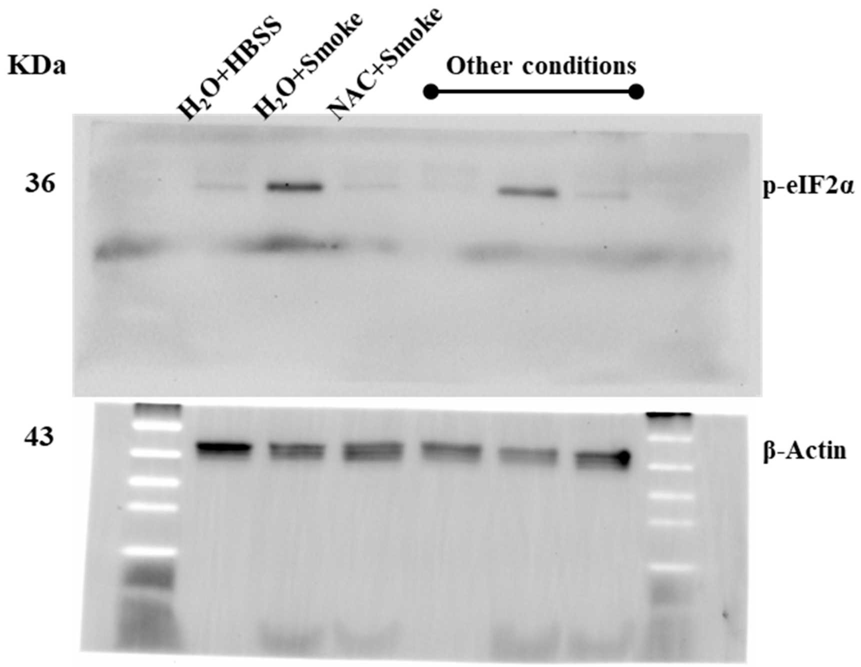

Figure S7: Reactive oxygen species induce smoke-associated eIF2α phosphorylation in CFBE cells. Immunoblotting captures a 7-fold increase in p-eIF2α abundance following smoke extract exposure (See Figure 8A,C in manuscript). Pre-treatment with the reactive oxygen species scavenger N-acetylcysteine (NAC, 5 mM, 15 min) prior to smoke exposure fully abrogates smoke-induced p-eIF2α increase. These ratios are calculated by normalizing to both p-eIF2α levels under H_2O+HBSS exposure conditions and β-Actin levels.

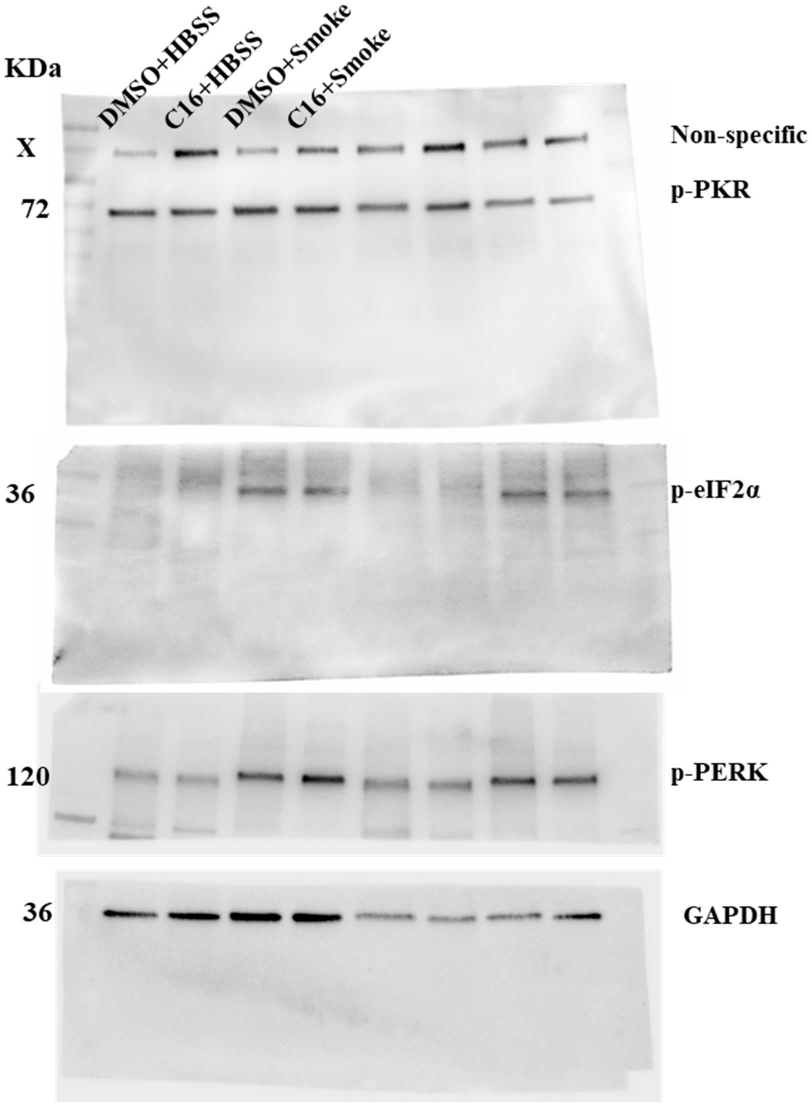

Figure S8: Smoke extract exposure does not induce PKR phosphorylation in CFBE cells. CFBE cells were pre-treated or not with C16, a specific PKR inhibitor, prior to their exposure to 15% smoke extract for 2h. Immunoblotting was performed to measure p-PKR, p-eIF2α and p-PERK levels. Phospho-PKR antibody targeting both Thr446 and Thr451 phosphorylation sites was used in this experiment. Immunoblotting showed that p-PKR level did not increase beyond the basal level in response to smoke exposure. Antibodies targeting Thr446 phosphorylation site alone did not show any basal p-PKR signal under control or smoke exposure conditions (data not shown). Additionally, inhibiting PKR using C16 didn't influence p-PKR level or halt the increase in p-eIF2α or p-PERK levels strongly suggesting that PKR doesn't activate the ISR in CFBE cells in response to smoke exposure.

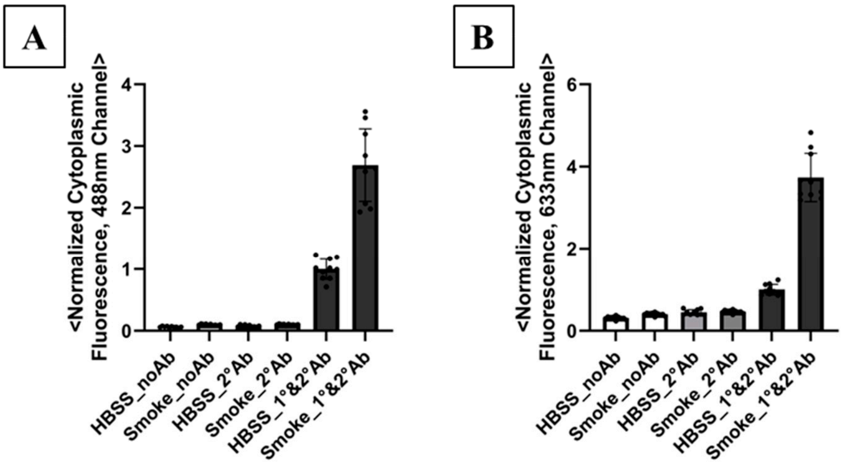

Figure S9: Quantification of the contribution of mean cytoplasmic autofluorescence and non-specific binding to mean cytoplasmic G3BP1 and p-eIF2α fluorescence intensity. CFBE cells were exposed to 15% HBSS or smoke extract for 2h. Immunofluorescence (IF) was performed in cells processed without primary (1°Ab) and secondary (2°Ab) antibodies, with secondary antibodies alone, or with primary and secondary antibodies. Cells' nuclei were stained with DAPI under all conditions to guide the nucleus-based image quantitative analysis. Cells were imaged under the same imaging conditions. The mean cytoplasmic signal was computed using the nucleus-based image analysis. Images of cells processed without primary and secondary antibodies were used to measure mean cytoplasmic autofluorescence (noAb). Images of cells processed with secondary antibodies alone were used to measure mean cytoplasmic autofluorescence combined with secondary antibody non-specific binding contribution (2°Ab). Images of cells processed with both primary and secondary antibodies were used to measure mean cytoplasmic fluorescence encompassing specific p-eIF2α fluorescence and background fluorescence (1°&2°Ab). Mean cytoplasmic fluorescence under all conditions was normalized to mean cytoplasmic fluorescence of G3BP1 (A) or p-eIF2α (B) under HBSS exposure condition. (A) The mean cytoplasmic fluorescence intensity collected in the 488 nm excitation channel (corresponding to G3BP1) was computed under HBSS and smoke conditions. Based on the quantitative analysis, mean cytoplasmic autofluorescence (noAb) constituted ~8% of mean G3BP1 fluorescence signal under HBSS exposure condition and in the presence of both primary and secondary antibodies (1°&2°Ab), while mean autofluorescence combined with the contribution of non-specific binding of the secondary antibody (2°Ab) constituted 9%. Smoke exposure did not change the mean autofluorescence or non-specific binding contributions. (B) The mean cytoplasmic fluorescence intensity collected in the 633 nm excitation channel (corresponding to p-eIF2α) was computed under HBSS and smoke conditions. Based on the quantitative analysis, mean cytoplasmic autofluorescence (noAb) constituted ~35% of mean p-eIF2α fluorescence signal under HBSS exposure condition and in the presence of both primary and secondary antibodies (1°&2°Ab), while mean autofluorescence combined with the contribution of secondary antibody non-specific binding (2°Ab) constituted ~45%. Smoke exposure did not change the mean autofluorescence or non-specific binding contributions. Knowing that 45% of mean cytoplasmic p-eIF2α fluorescence signal measured under HBSS conditions is related to autofluorescence and nonspecific binding and that smoke does not change this contribution, that helps to adjust the increase in mean p-eIF2α fluorescence levels under smoke exposure condition from 3.7-fold to 6-fold above that under HBSS treatment condition. This is accomplished by subtracting an equal value of 0.45 of the normalized p-eIF2α fluorescence signal under HBSS and smoke conditions. This adjusts p-eIF2α fluorescence level under HBSS to 0.55, while under smoke exposure conditions, p-eIF2α fluorescence reads 3.25. The p-eIF2α level under smoke exposure condition then increases by 5.9-fold over its level under HBSS condition.
